# A Systematic Review of Closed Head Injury Models of Mild Traumatic Brain Injury in Mice and Rats

**DOI:** 10.1089/neu.2018.6127

**Published:** 2019-05-22

**Authors:** Colleen N. Bodnar, Kelly N. Roberts, Emma K. Higgins, Adam D. Bachstetter

**Affiliations:** ^1^ Department of Neuroscience, University of Kentucky, Lexington, Kentucky.; ^2^ Spinal Cord and Brain Injury Research Center, University of Kentucky, Lexington, Kentucky.

**Keywords:** animal models, common data elements, concussion

## Abstract

Mild TBI (mTBI) is a significant health concern. Animal models of mTBI are essential for understanding mechanisms, and pathological outcomes, as well as to test therapeutic interventions. A variety of closed head models of mTBI that incorporate different aspects (i.e., biomechanics) of the mTBI have been reported. The aim of the current review was to compile a comprehensive list of the closed head mTBI rodent models, along with the common data elements, and outcomes, with the goal to summarize the current state of the field. Publications were identified from a search of PubMed and Web of Science and screened for eligibility following PRISMA guidelines. Articles were included that were closed head injuries in which the authors classified the injury as mild in rats or mice. Injury model and animal-specific common data elements, as well as behavioral and histological outcomes, were collected and compiled from a total of 402 articles. Our results outline the wide variety of methods used to model mTBI. We also discovered that female rodents and both young and aged animals are under-represented in experimental mTBI studies. Our findings will aid in providing context comparing the injury models and provide a starting point for the selection of the most appropriate model of mTBI to address a specific hypothesis. We believe this review will be a useful starting place for determining what has been done and what knowledge is missing in the field to reduce the burden of mTBI.

## Introduction

Mild traumatic brain injury (mTBI), caused by blunt trauma, acceleration, or deceleration forces, is a significant public health concern.^1^ In the United States, mTBI is estimated to occur in 1.6–3.8 million cases annually.^2^ Estimates of mTBI are under-reporting cases of mTBI attributed to the fact that many individuals who sustain a mTBI never seek medical treatment.^3^ Not only are incidences of mTBI on the rise— 62% increase in recreation-related mTBI cases over a 10-year period^4^—there is also a growing appreciation that a mTBI is not benign and the brain may not fully recover from the injury with time. The Department of Veterans Affairs and the Department of Defense Clinical Practice Guidelines and the World Health Organization guidelines classify a head injury as a mTBI according to the following criteria: normal structural imaging; loss of consciousness <30 min; alteration of consciousness less than 24 h; post-traumatic amnesia of less than a day; and an initial Glasgow Coma Scale of 13–15.^5^ By far, the majority of mTBIs are caused by a closed head injury.^6^ The Centers for Disease Control and Prevention (CDC) concluded there was the need for research to understand the full magnitude of mTBI incidence, risk factors, and strategies to reduce and improve mTBI outcomes.^1^ Much of this research begins with understanding the pathological and mechanistic changes of mTBI in pre-clinical models.

The use of animal models in TBI research is crucial. Many animal models have been developed over the last 80 years to replicate the different unique features of mTBI (e.g., emotional or cognitive symptoms), as well as biomechanical forces (e.g., impact or rotational). Although the overall number of experimental mTBI studies are small in number, they are steadily increasing at a rate reflecting the public's appreciation of the seriousness of mTBI. However, to paraphrase an expert in animal models of TBI, the field is a “wild west”; referring to the vast array of injury methods currently used to create a rodent model of mTBI. The aim of this systematic review is to compile a comprehensive list of TBI rodent models that are specific to mild closed head injury. Our review includes information about the methodology as well as the broad classes of outcomes. Understanding all of the different models, as well as pathologies associated with the model, is needed to propel the field forward by taming the wild west, building a framework of common data elements for future reporting of mTBI models, and uncovering gaps in our current knowledge base.

## Methods

### Search criteria

**FIG. 1.**
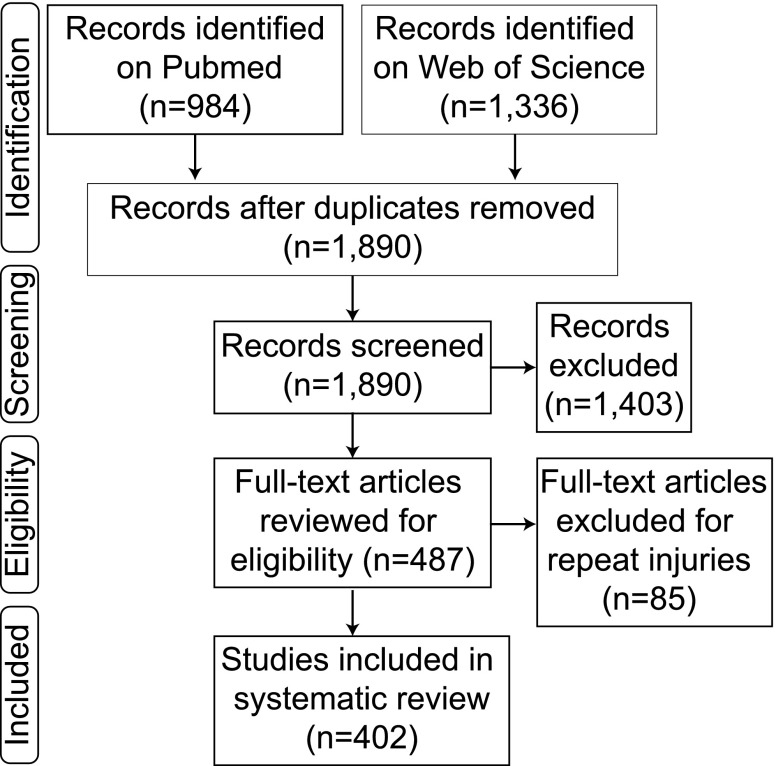
Methods flow chart. Identification through searches on two separate web-based platforms yielded 1,890 articles which were screened by abstract and then eligibility was determined via full text examination to exclude 1,403 articles. Removing mTBI articles in which repeat injuries were sustained, a total of 402 single mTBI articles were included in our review. mTBI, mild traumatic brain injury.

Our search criteria were established to be specific for mTBI models caused by a closed head injury. Following guidelines established by PRISMA,^7^ comprehensive searches (on May 15, 2018) of both PubMed and Web of Science were conducted using the following keyword search: mild TBI, concussion, closed head injury, rodent, mice, mouse, or rat; excluding controlled cortical impact, fluid percussion, or review articles. Using the advanced search tools on PubMed and Web of Science, both title and abstract were searched with the following Boolean search strategy: [(((((((rodent) OR rat) OR mouse) OR mice)) AND (((mild TBI) OR concussion) OR closed head injury))) NOT ((CCI) OR fluid percussion)]. From PubMed, 984 articles were given in the final results, and Web of Science produced 1336 articles. Both of these lists of articles were combined and duplicate references were removed, leaving 1890 articles (Fig. 1).

### Inclusion/exclusion criteria

Abstracts and titles were screened by C.N.B. to include only peer-reviewed primary research reports specific for mild, closed head TBI only in rodents. All other types of articles were excluded. Blast injuries were excluded because, although military related blast injuries are a major cause of mTBI,^5^ the vast complexity of the different injury models used were beyond the scope of this review.^8^ All repeat injury models (85) were also considered beyond the scope of this review and were thus excluded (Fig. 1).

### Retrieval of information from full-text articles

For collection of information on methods of each of these articles, a GoogleForm was used by C.N.B., K.N.R., and E.K.H. The title, first author, last author, publication date, name of model, and references cited for the model were collected as general identifiers. For the method of injury induction, the following information was collected: injury device used, anesthesia use, surgery indicator, injury device type, head fixed with method of fixation, animal stabilization method, impact tip size, impact tip shape, impact tip material, impact surface, impact location, weight for drop, height for drop, tube composition, and the type of impact absorbent materials used (if any). Species, sex, and age of animals were also recorded. Finally, injury outcomes were collected, including mortality rate, righting reflex latency, neuroscore, motor deficits (open field, balance beam, rotarod, etc.), cognitive deficits (novel object, Morris water maze, radial arm water maze, Y/T maze, etc.), affective behavior deficits (elevated plus, social, sucrose preferences, etc.), and histology (cell and tissue changes, axonal injury markers, myelin markers, gliosis markers, etc.). Information on outcome variables was collected only between sham and control animals, not with any treatments done within the publication. If any treatments were done in the publications, the effects observed with treatment were not considered as content for this review.

## Results

From searches on both PubMed and Web of Science, a total of 1890 articles were initially included. From this list, articles were screened for inclusion and exclusion criteria (Fig. 1). From this screening, the following articles were excluded: not including a TBI (*n* = 293); any article in which the skull was open and the brain surface was impacted (controlled cortical injury [CCI]/fluid percussion injury/or open skull; *n* = 125); any injury that was reported as moderate or severe (*n* = 237); duplicates missed in the original removal (*n* = 21); non-rodent models (*n* = 54); articles that used an *in vitro* or computational method (*n* = 63); blast injuries (*n* = 160); book chapters and reviews that were missed in the initial identification (*n* = 96); articles in another language (*n* = 24); articles with no explanation of their method, no sham animals, or not a full article (*n* = 20); and repeat injuries (*n* = 85). Finally, 402 articles were determined to be single, mild, TBIs to rodents. These 402 articles were then examined in full text and the common data elements were compiled (Fig. 1).

**FIG. 2.**
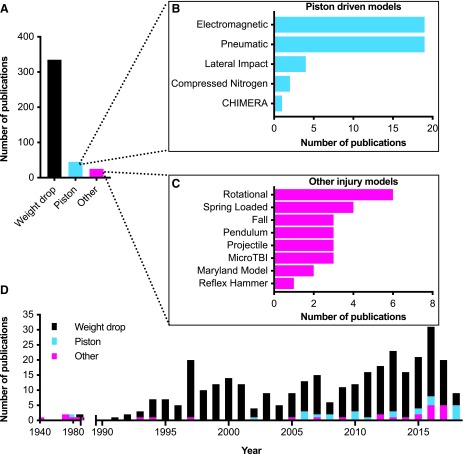
Overall summary of included studies. Of the 402 articles included in our final analysis, 3 major categories of models were found (**A**). Within the piston category (**B**) and the “other” models (**C**) there was considerable variability. A breakdown by year of publication (**D**) demonstrated the weight drop model over the last four decades and the increase in use of piston driven models over the last decade. TBI, traumatic brain injury. Color image is available online.

Of the 402 articles identified three main groups of injury models emerged. The largest group was weight drop models (*n* = 335),^9–343^ followed by piston-driven models (*n* = 43),^344–386^ and then all “other” models (*n* = 25)^387–411^ (Fig. 2A). Please note that one article used both a weight drop model and a piston-driven model and was thus included in both of these groups.^199^ Within the piston-driven models and the “other” models, there was a wide variety of methods used (Fig. 2B,C). We compared how often the different categories of models were used over time to identify trends in usage (Fig. 2D). Weight drop models have featured prominently in the literature since the early 1990s. Piston-driven models gained popularity beginning in 2002. The “other” models were typically used early (1941–1987) and used more unconventional methods to induce mild brain injury. Once the weight drop models became more popular in the 1990s, this model became the dominant model in use during this time. Beginning in the mid-2000s, new models besides weight drop began to emerge, each attempting to model different aspects of mild TBI and increase the reproducibility of the injury model. At the same time, the number of weight drop publications plateaued.

From each article, the time post-injury at which the dependent variables were measured was collected (up to 1 day, up to 1 week, up to 1 month, over 1 month, or over 1 year). A vast majority were recorded under a month, with 39% reporting up to 1 day post-injury, 35% reporting up to a week after injury, and 19% reporting up to 1 month after mTBI (Supplementary Appendix [SA] 0.1). Only 24 articles reported dependent variable measurements over a month, and only two publications reported measurements after a year (SA 0.1) (see online supplementary material).

### Weight drop model

**FIG. 3.**
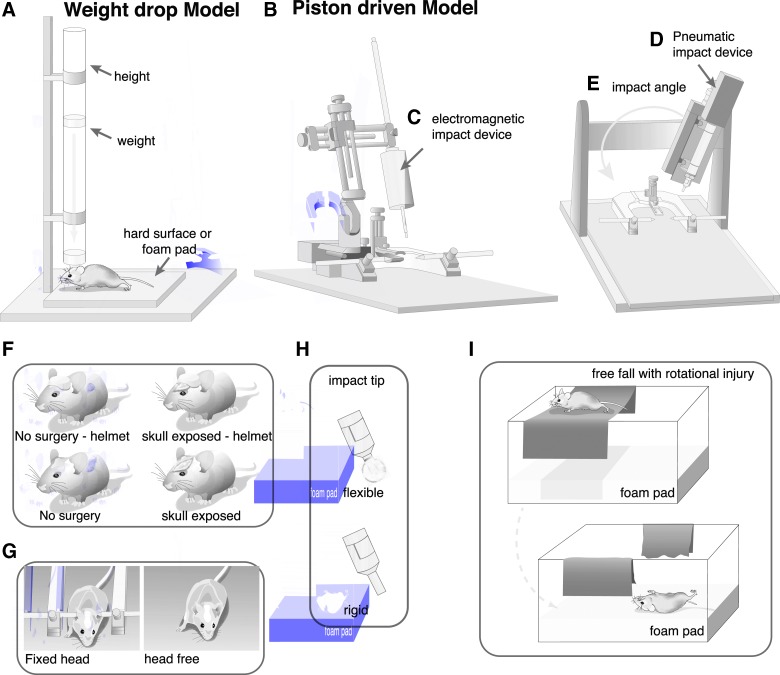
Example experimental set-up for weight drop and piston mTBI models. In the weight drop model (**A**), a variable weight is dropped from a variable height, onto the head of the animal and the animal can be on either a hard surface or a foam pad. Piston driven models (**B**), use either an electromagnetic (**C**) or a pneumatic (**D**) driven piston that is set to a specified velocity and impact depth and strikes the head of the animal. In piston driven models, the impact angle (**E**) can vary between study designs. In both the weight drop and the piston driven models the impact surface can vary with either a helmet or no helmet on the intact scalp or the exposed skull (**F**). Further, the head can be either fixed or free to rotate after the impact (**G**). The impact tip which contacts the head of the animal to induce injury can be either flexible or rigid (**H**) causing different injury biomechanics. An emerging model utilizes rotation following impact by placing the animal on a thin sheet following impact the animal falls through the sheet onto a foam pad (**I**). Color image is available online.

**FIG. 4.**
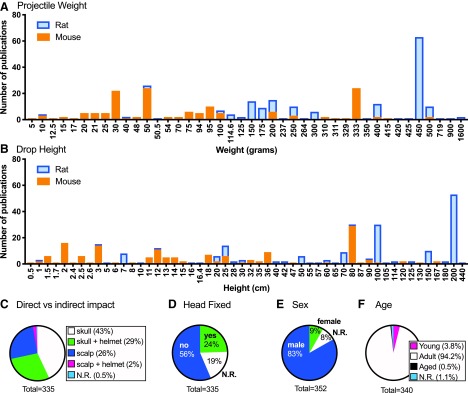
Common data elements for the weight drop model. Weight drop models have been used with a variety of different weights (**A**) and heights (**B**). Mice were injured typically using lower weights and heights, while rats were injured using heavier weights and heights. Injuries were induced on different surfaces (**C**) with the head either fixed or unfixed (**D**). A majority of papers used male (**E**), adult animals (**F**). Total numbers for (E) and (F) are greater than the 335 total publications because several publications reported using more than one sex or age of animals. N.R., not reported. Color image is available online.

The weight drop model consists of dropping a projectile of specified characteristics through a tube at a specified height onto the head of the animal (Fig. 3A). We found considerable variation in the reported weight of the projectile (Figs. 3A and 4A) and drop height of the projectile (Figs. 3A and 4B) between different publications. Additional model-specific common data elements that we captured and varied between different publications included: 1) if the mice were anesthetized at the time of impact; 2) if surgery was performed; 3) direct versus indirect impact to the skull (Figs. 3F and 4C); 4) impact location; 5) if the head was immobilized (Figs. 3G and 4D); 6) the surface the animal was placed on (Fig. 3A,H); 7) projectile shape; and 8) projectile material. Animal-specific common data elements that we captured included 1) sex of the animals (Fig. 4E) and 2) age of the animals (Fig. 4F).

#### Weight drop model: animal-specific common data elements

The reported animal-specific common data elements for the weight drop method showed remarkably little variation in sex or age of the animals used. The use of rats and mice occurred at an approximately equal ratio—57% used rats (SA 1.1), 43% used mice (SA 1.2), and two publications used both rats and mice (SA 1.3). In the 8% of publications that used female animals (SA 1.4), 17 of those articles used both males and females and only eight analyzed data by sex for sex differences (Fig. 4E; SA 1.5). Adult animals were the most common age reported (Fig. 4F; SA 1.7). At the young age range, P7 was a typical age used for mice, whereas P17–48 was common for rats (SA 1.6) (see online supplementary material). Only two publications specifically reported using aged mice, as defined by the authors of the publication (SA 1.8).

#### Weight drop model: model-specific common data elements

Our review of the model-specific common data elements for the weight drop method was informative for both the intermodel similarities, but also for the degree of variations that have been used. For 98% of the publications using the weight drop method, animals were anesthetized at the time of the impact, leaving six publications that did not use anesthesia (SA 1.9). The majority (72%) of articles created an incision to expose the skull (SA 1.10). Of the publications that exposed the skull, metal disc helmet was placed on the skull in 40% of publications to diffuse the blow and reduce skull fractures and focal lesions (Figs. 3F and 4C; SA 1.11). The scalp was the surface of impact in 26% of articles (SA 1.12) (see online supplementary material), and a helmet on the scalp was used in 2% of articles (SA 1.13). There was an even split on the location of the injury, with 49% reporting a midline injury, typically located in the midcoronal plane between bregma and lambda on the sagittal suture, and 48% reporting a lateral injury, typically located 1–2 mm lateral of midline in the midcoronal plane. In 56% of publications, the head was not secured and 19% of publications did not report this essential common data element (Fig. 4D). In 24% of publications, the head was held in place by methods that ranged from the heads being fixed rigidly with ear bars in a stereotactic frame (35%; SA 1.14), loosely between two blocks (6%; SA 1.15), or within the hand of the experimenter (17%; Fig. 3G; SA 1.16). (see online supplementary material). Similarly, 53% of publications did not report what the animal's head was resting on when it received the impact. Of those that reported resting surface of the head, a foam pad was used in 79% of publications (SA 1.17). We noted that few publications described the type and source of the foam pad, the spring constant of the foam, or how often it was replaced. Other surfaces that animals were placed on included, but were not limited to, foil (SA 1.18) see online supplementary material at (http://www.liebertpub.com), rubber (SA 1.19), or spring-loaded platforms (SA 1.20). Fifty-three percent of weight drop publications did not specifically describe the surface that the animal rested on during injury induction.

The two major weight drop–specific common data elements where we observed significant variability was in the weight of the projectile, and the projectile drop height (Fig. 4A,B). The majority of publications that reported impactor material used a rigid projectile, which was most often a brass weight (75%; Fig. 3I; SA 1.21). Whereas 25% of publications used a non-rigid projectile, which was most often a silicone tipped metal rod (SA 1.22), a few publications reported using a specific diameter projectile, but most did not report this common data element. Of the 57% of the total articles that used rats, the weight of the projectile used was most often 400 g or heavier (Fig. 4A). Also, projectiles were dropped from greater heights, on average, in the studies that used rats compared to experiments with mice (Fig. 4B). Seventy-six publications cited Marmarou and colleagues for the methods of the weight drop model.^179^ The investigators used a few variations on the name of the model (e.g., Marmarou, modified Marmarou, or impact-acceleration injury model), but most of the common data elements associated with the model were consistent. For instance, a 450-g brass weight was dropped from a height of 200 or 100 cm. The use of a 450-g weight with a 100-cm height was reported in 13 publications (SA 1.23), whereas a 200-cm drop height was reported in 44 publications (SA 1.24). Another aspect associated with these 57 publications was the use of a steel disk placed midline on the exposed skull as a “helmet.” Animals were anesthetized and often intubated. The head was generally free to move, but was supported on a foam pad.

We found a cluster of publications in mice (*n* = 29) that used a drop height of 80 cm (SA 1.25). Of these 29 publications, 76% used a 30-g metal weight, commonly with a lateral impact (79%). In these 29 articles, the impact was most often (83%) to the scalp. A helmet was used in one publication.^380^ A second cluster of 32 reports (22% of mouse publications) used a drop height of 2.5 cm or less (SA 1.26). Of these 32 reports, 47% used a 333-g projectile (SA 1.27), 75% induced a lateral injury to one hemisphere of the brain (SA 1.28), and 88% delivered the impact directly to the skull (SA 1.29). Most of these (23 of 32) articles were referencing Flierl and colleagues and/or Chen and colleagues for the methods.^85,412^

We also identified a few additional model-specific common data elements that were associated with either preventing rebound impacts or to cause a rotational injury. Specifically, some publications report methods to prevent rebound impacts from the projectile, including attaching a rope to the projectile (SA 1.30); however, a majority of the publications did not report this common data element. There are 12 publications of a modified version of the weight drop model where the animals are placed either on a Kimwipe or a piece of scored tin foil. Placement of the mice on the non-rigid material allows the animal to fall through the material and lands on a foam pad underneath from the force of the projectile impacting the head (Fig. 3H; SA 1.31).

#### Weight drop model: injury-induced functional and histopathological changes

In addition to the animal- and weight drop–specific common data elements, we also collected information about the previously assessed functional and histological endpoints in the model. We collected data for the presence or absence of an injury-induced change only. It was beyond the scope of this review to describe specifics of when the injury-induced changes occurred, if they resolved with time, and which endpoints showed the most significant injury effect. Our goal in summarizing the common endpoint measurements was to identify regularly reported endpoints that would be useful for comparisons between studies and highlight areas that are understudied.

#### Weight drop model: motor skills assays

The Neurological Scale Score (NSS) is a measurement used to test sensorimotor skills and involves a battery of tests.^379^ This test has been used and described for a mild (<10 points), moderate (11–14 points), or severe injury (>14 points).^48^ From our search, we found that of the 335 weight drop publications, only 31% reported doing the NSS. Of these articles, 87% reported a deficit and 13% reported no deficits after mTBI (SA 1.32). Further, righting reflex, a measure that is commonly used as a surrogate for loss of consciousness,^5^ was only reported in 10% of articles (SA 1.33). The most common latency for righting reflex was between 1 and 10 min (81% of those who reported). Mortality rate was reported in 24% of publications. Low mortality (0–5%) was found in 39% of these articles (SA 1.34), a moderate mortality (5–30%) was found in 51% of articles (SA 1.35), and a high mortality rate (30%+) was found in 10% of those articles who reported (SA 1.36).

**FIG. 5.**
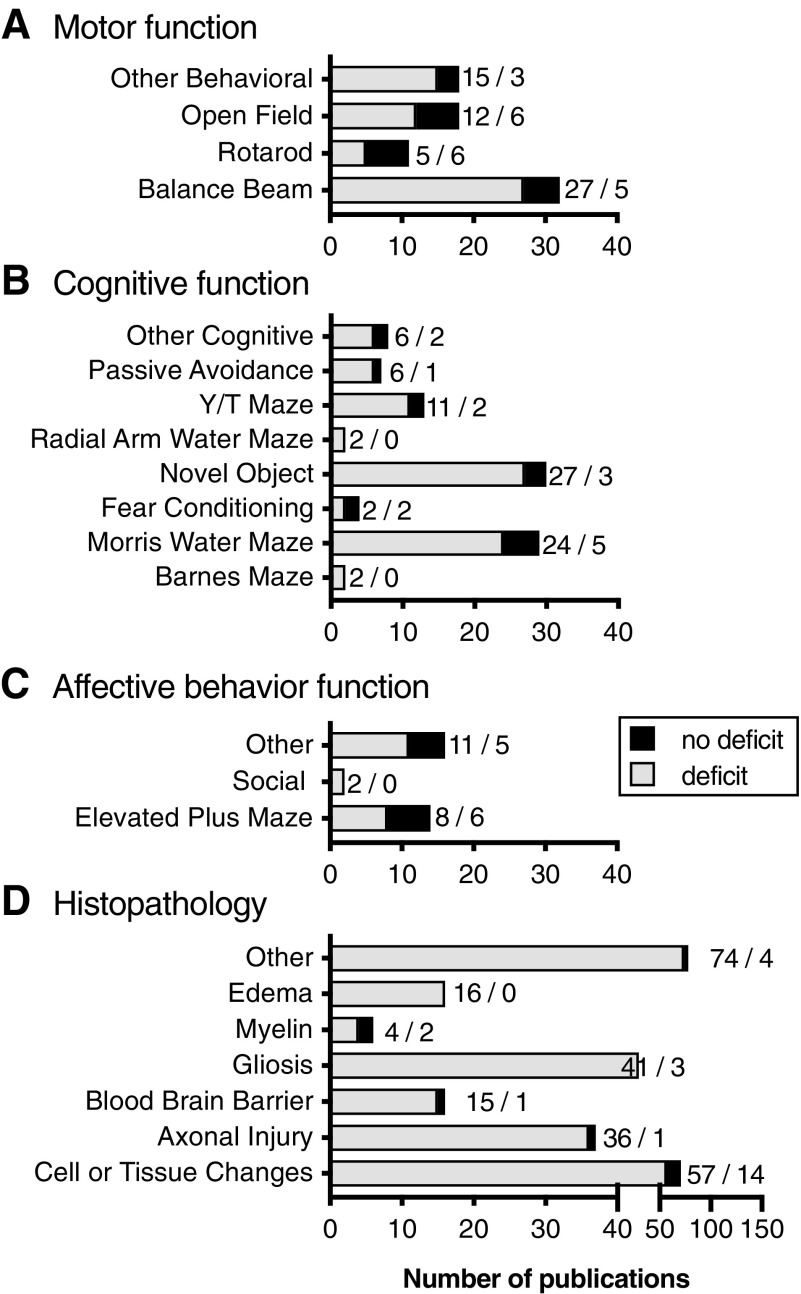
Functional and Pathological deficits observed in the weight drop models of mTBI. Outcome measures collected included motor function (**A**), cognitive function (**B**), affective behaviors (**C**), and histology measures (**D**). Numbers indicate the number of studies with / without deficits. mTBI, mild traumatic brain injury.

An assessment of motor skills was most often done by one of three tests—balance beam, rotarod, and open field—and reported in 19% of weight drop publications (Fig. 5A). Specifically, the balance beam test was reported in 50% of publications with a deficit in 84% of those studies (SA 1.37). In the open field and rotarod assay, 67% (SA 1.38) (see online supplementary material) and 45% (SA 1.39) of the studies found a deficit, respectively. In addition to these three tests, nine additional motor skills tests were measured, including: foot placement (4/0), grid walking (1/0), grip test (3/1), tape removal (1/0), general activity (2/1), staircase test (0/1), whisker test (2/0), seizure susceptibility (1/0), and thermal or mechanical withdrawal (1/0; *n* = deficit/no deficit; SA 1.40).

#### Weight drop model: learning and memory assays

Cognitive tasks typically involve learning in some capacity on the part of the animal, and common tests we found included passive avoidance, Y/T maze, radial arm water maze, novel object recognition, fear conditioning, Morris water maze, and Barnes maze. Twenty-one percent of weight drop articles reported doing some sort of cognitive testing. Of the articles reporting cognitive testing, deficits were recorded in 84% of cases (Fig. 5B). All of the publications that used either the Barnes maze or the radial arm water maze found deficits after mTBI (SA 1.41–42). In the Y/T maze (85%; SA 1.43), novel object (90%; SA 1.44) (see online supplementary material), and Morris water maze (83%; SA 1.45), a majority of publications found a weight drop–induced deficits. When fear conditioning was tested, 50% found deficits (SA 1.46). Passive avoidance was reported in two publications, and both found deficits (SA 1.47). Other cognitive testing identified, included the water finding test (2/2), location discrimination test (1/0), go/no-go testing (1/0), closed circle exiting (1/0), and novel context mismatch (1/0; *n* = deficit/no deficit; SA 1.48).

#### Weight drop model: affective behavior testing

Affective behavior tests are designed to be used as a surrogate for more complex emotions like anxiety (and other affective behaviors). Of the 355 articles that used the weight drop method, only 7% reported affective behavior tests. The elevated plus or zero maze and social testing were commonly used in these publications (Fig. 5C). In the two publications that tested social behaviors, both found deficits compared to sham (SA 1.49). In the elevated plus maze tests, eight publications found deficits whereassix other publications did not find any deficits (SA 1.50). Other affective behavior tests that were reported included temperature sensitivity (1/0), tail suspension (1/2), olfactory avoidance (1/0), nociception (1/1), forced swim (6/1), fear conditioning (0/1), and acoustic startle reflexes (1/0; *n* = deficit/no deficit; SA 1.51).

#### Weight drop model: histopathology

In addition to behavioral tests, we collected information on which articles reported common histological changes that have been cited in the literature on brain injury. Cell or tissue changes are typically looked at by hematoxylin and eosin, cresyl violet, neuronal nuclei, or Nissl staining. Other categories of histological interest included axonal injury (shown by amyloid precursor protein, silver staining, or neurofilament stains), gliosis (shown by glial fibrillary acidic protein, ionized calcium binding adaptor molecule 1, CB68, or CD11b staining), myelin changes (shown by luxol fast blue or myelin basic protein staining), and blood–brain barrier disruption (Evans blue). Other staining was noted even if it was not within these categories.

Histology was more commonly reported as compared to behavioral changes in the articles included in our final analysis of weight drop models. Of the 335 articles, 55% reported using histology of these major categories and, of those, 88% found there to be a significant change after mTBI compared to sham animals (Fig. 5D). Changes to cellular and tissue makeup were reported as significantly different from sham in 80% of publications whereas 14 publications did not find a deficit (SA 1.52). Deficits were also more common in axonal injury (95%; SA 1.53); blood–brain barrier disruption (94%; SA 1.54); edema (100%; SA 1.55); gliosis (93%; SA 1.56); and myelin staining (67%; SA 1.57). Many other histological measures were done for specific proteins of interest for the experimental design. A few of these include terminal deoxynucleotidyl transferase dUTP nick end labeling or apoptosis staining (11/1), inflammation/immune activation (10/0), caspase activation (3/0), complement activation (3/0), as well as many others (SA 1.58).

### Piston-driven closed head injury models

Injuries caused by a piston are typically induced by zeroing the piston on the surface of the skull or scalp and then delivering an injury at a specific depth, velocity, or impact force (Fig. 3B). There are a few variations of the piston devices (Fig. 2B), including compressed nitrogen, electromagnetic, or pneumatically driven pistons (Fig. 3C,D). Another variation within this category is location of the injury and placement of the animal. For example, in the CHIMERA model, the animal is placed on its back within the device, and the injury is induced from below, thus allowing the head to flex after impact.^202^ Another example of using a piston at a different injury location is the “Hit & Run” model.^400^ In order to induce an injury using this particular model, the animal is hung from a string by the incisors, allowing a piston to strike the side of the head. This approach allows the animal to freely move after the impact. Other models use lateral angle for impact; however, in these models, animals lie on a flat surface and move laterally after the impact^106,199,236^ (Fig. 3E).

**FIG. 6.**
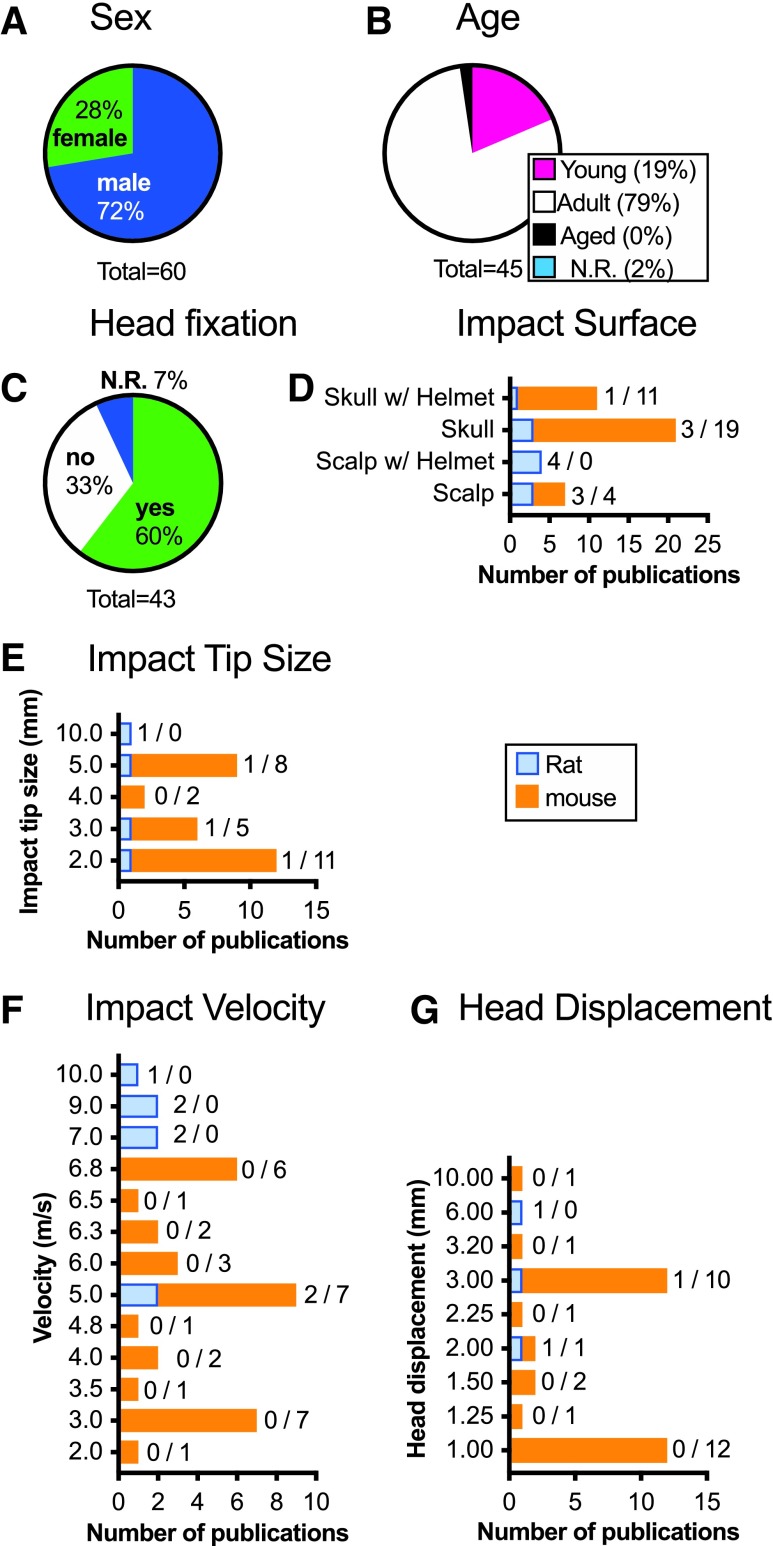
Piston driven CHI models of mTBI common data elements. Most animals were male (**A**) and adult (**B**). The head was most often fixed (**C**) and the impact surface varied (**D**). The impact tip size when reported was typically 5 mm or less (**E**). Impact velocity (**F**) and depth (**G**) were smaller for mice than rats. Numbers indicate the number of studies using rats / mice. Total numbers are greater than the 43 total publications because several publications reported using more than one sex or age. CHI, closed head injury; N.R., not reported. Color image is available online.

**FIG. 7.**
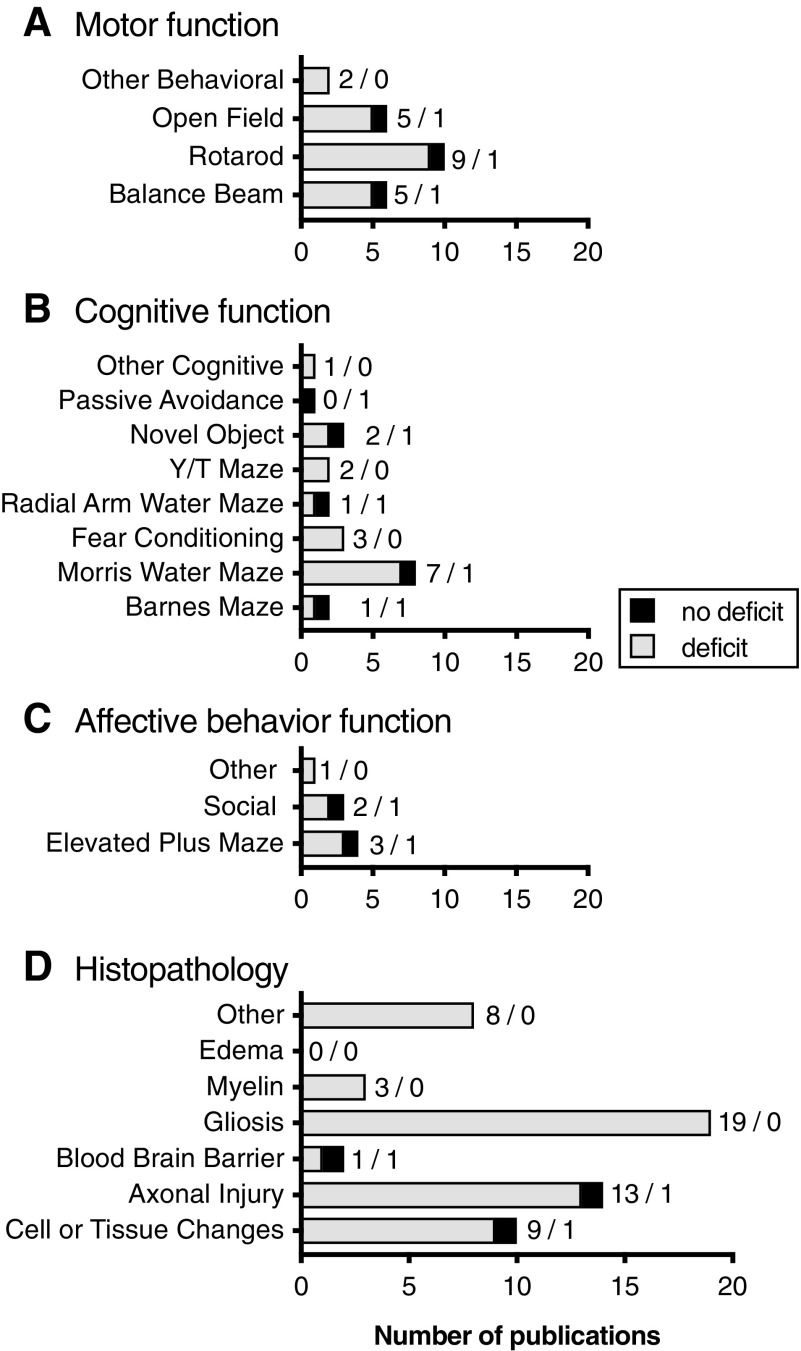
Outcome measures for piston driven injury models. Common tests typically reported for motor function (**A**), cognitive function (**B**), affective behaviors (**C**), and histological measures (**D**) were reported. Numbers indicate the number of studies with / without deficits.

As in the weight drop articles, we also collected animal indicators such as 1) species, 2) sex (Fig. 6A), and 3) age (Fig. 6B). Common data elements of the injury were collected for these piston-driven models as well. We recorded 1) anesthesia use; 2) surgery indication; 3) head fixation (Figs. 3G and 6C); 4) method of head fixation; 5) impact location; 6) impact surface (Figs. 3F and 6D); 7) what material the animal was placed on; 8) impactor tip size (Fig. 6E) and shape; 9) impact velocity (Fig. 6F); and 10) head displacement (Fig. 6G).

#### Piston-driven closed head injury models: animal-specific common data elements

Most articles using piston-driven injury utilized mice (74%) as opposed to rats (26%; SA 2.1). Of the articles that reported the sex of the animals, males (72%) were most common (Fig. 6A; SA 2.2). When females were used, 94% of the articles also included males; and only half of the time were data separated by sex to determine sex differences (SA 2.3). Adult animals were most prevalent (79%), followed by pups (19%; SA 2.4), with no studies reporting the use of aged animals (Fig. 6B).

#### Piston-driven closed head injury models: model-specific common data elements

Anesthesia was uniformly used for this injury model. Direct impacts were given either to the scalp (17%; SA 2.5) or exposed skull (49%; Fig. 6D; SA 2.6) (see online supplementary material). A helmet was used in 35% of articles, either placed on the scalp (SA 2.7) or skull (SA 2.8). The head was left free to move after the injury in 33% of the publications (Fig. 6C; SA 2.9), and in some cases the animal was placed on a foam pad (SA 2.10) or on scored tin foil and allowed to fall through the foil after the injury (Fig. 3H; SA 2.11). Generally, if an animal's head was fixed, a stereotactic frame was used (77%; Fig. 3G; SA 2.12). Other methods of securing the head varied in the amount that the head was able to move after injury; this included foam wrapped ear bars,^355^ an acrylic or resin mold,^347,363^ a plastic collar,^348^ a mouse restrainer,^349^ or secured vertically from the incisors.^400^

Velocity of the impact used in mice was typically slower as compared to rats (Fig. 6F). Six publications used an impact velocity of 6.8 m/s, the fastest velocity used in mice (SA 2.13). All six cited the previous works by Lynch and colleagues.^365,366^ With this model, a rigid, flat impact tip of 2 mm was typically used (Fig. 6E). An impact of 5 m/s was reported in seven publications in mice with a 5-mm impact tip and impacted either 1 or 1.5 mm deep (SA 2.13). A common reference was not used for this configuration. Publications using rats (*n* = 11) reported using faster velocities of ranging from 5 to 10 m/s (SA 2.14).

The depth of impact set on the piston device was also typically smaller for mice than rats (Fig. 6G). One head displacement depth of 10 mm was used in the Hit & Run model.^400^ All other reported head displacement depths were 3.2 mm or less (SA 2.15).

Different materials could be used within the impactor tip, with 58% using a rigid tip and 5% reporting a flexible tip such as rubber or silicone (Fig. 3I; SA 2.16). A large number of publications did not report the material of the impact tip (37%; SA 2.17). The shape of the impact tip was most often flat (60%) or round (35%) when reported (SA 2.18).

#### Piston-driven closed head injury model: injury-induced functional changes

We found for the piston-driven model that mortality was under-reported (only 23% of articles reporting), with most (90%) reporting a mortality rate of 0–5% (SA 2.19). Moderate mortality was reported in one publication, and no publications reported high mortality (SA 2.19). Righting reflex was reported in 33% of the piston-driven closed head injury (CHI) models, with most having a latency of 1–10 min (79%; SA 2.20).

#### Piston-driven closed head injury model: motor skills assays

The NSS was not commonly reported in the piston-driven CHI model, with only one publication reporting no deficit^365^ and two finding a significant change from sham after injury.^202,354^ Deficits were found in five publications after piston-driven injury in the balance beam, with one publication finding no deficits (SA 2.21). Rotarod was tested in 10 publications, and nine found deficits with one publication finding no change after injury (SA 2.22). Open field was tested in six publications, and in five, deficits after mTBI were found and one found no deficit (SA 2.23). Other motor tests assessed in the piston-driven CHI included wheel running and wire hanging, with deficits observed in both assays (Fig. 7A; SA 2.24).

#### Piston-driven closed head injury model: learning and memory assays

Forty-seven percent of piston-driven CHI publications reported cognitive testing. The Morris water maze was the most commonly used cognitive test after the piston-driven CHI, and deficits were commonly found (88%; SA 2.25). All three of the publications using fear conditioning after a piston-driven CHI reported an injury-induced deficit, whereas two of three publications using novel object recognition found an injury-induced deficit (SA 2.26). The Y/T maze (2/0), radial arm water maze (1/1), and Barnes maze (1/1) were each run in two publications (*n* = deficit/no deficit; SA 2.26). The passive avoidance test with no deficit and a labyrinth maze test with a deficit were reported only once (Fig. 7B; SA 2.26).

#### Piston-driven closed head injury model: affective behavior testing

Affective behavior changes in different affective behaviors were reported in 19% of the piston-driven models (*n* = 8). When these tests were conducted, three of four found deficits in the elevated plus maze (3/1; SA 2.27) and two of three found social deficits (Fig. 7C; SA 2.28). One other affective behavior test was conducted in this group, a sucrose preference task, in which a deficit was found after injury.^385^

#### Piston-driven closed head injury model: histopathology

Histopathology was reported in 74% of the piston-driven CHI publications we reviewed. Cell or tissue changes were reported in 9 of 10 publications (SA 2.29). Axonal injury was reported in 13 of 14 publications (SA 2.30). Gliosis was reported in 19 of 19 publications (SA 2.31). Three publications reported injury-induced alterations in myelin (SA 2.32). The other histopathological endpoints that we noted included blood–brain barrier alterations (1/1), inflammation (2/0), caspase activation (1/0), and complement activation (1/0; *n* = deficit/no deficit; Fig. 7D; SA 2.33).

### “Other” models

The last broad class of mTBI models we defined as “other” models. We organized these models based on the biomechanical mechanism or type of device used to cause the injury. As shown in Figure 2C, the most commonly used of these “other” models was the rotational injury models (SA 3.1), whereas the least widely used was the reflex hammer model.^390^ Although some of these models date back to the 1940s, it is still important to consider these older and less-used methods given that these studies are informative on how mild TBIs have been modeled over time. Our review of these models, which follows, will go from the most to the least used (Fig. 2C).

The group of “other” models with the most publications are associated with a rotational biomechanical mechanism. Two models have been developed that utilized rotation to induce mTBI. The Medical College of Wisconsin Rotational Injury model (SA 3.1) produced an injury by using a spring-loaded launching arm that strikes a moment arm. This moment arm causes the device to rotate with the animal's head in it, thus causing the injury. The head is fixed after induction of anesthesia, and no incision is made to the scalp. In a similar model, Rostami and colleagues induced a rotational injury in anesthetized rats by placing the animal with an exposed skull in the injury device and hitting a bar within the device to cause rotation of the animal's head (SA 3.1). Of the rotational injury models, two publications used females,^278,409^ whereas four used males.^375,388,389,401^ All six publications used adult rats. When reported, both models resulted in low mortality when reported.^278,388^ with long righting reflex latencies of over 10 min.^388,389^ NSS was performed in one publication, and no deficits were found after injury.^409^

Spring-loaded injury devices—used pre-1990s—are another method used to induce mTBI. One of these models uses a modified rat trap to induce injury.^247,406,407^ No anesthesia was used in this model, and the injury was induced onto the intact scalp of the rat. The injury used the spring-loaded rat trap with a modified knob on the end to hit the midline of the adult male rat head. Mortality with this model was reported in one publication between 0% and 5%^407^ and in another 5–30%,^247^ and when a righting reflex was reported, it was between 1 and 10 min.^247^ Another model used a coiled spring-loaded gun to induce mTBI.^210^ With this model, a helmet was secured to the skull of the adult male rats. Mortality in this model was 5–30%, with a righting reflex of 1–10 min.

Fall-type injury models have the animals fall onto an immovable surface.^378,392,413^ The authors of these models speculate that this model may more closely mimic certain head trauma cases in humans with the head moving and hitting a stationary object.^413^ These injuries were induced both with and without anesthesia, but always to the scalp. The injury was induced when the animal's head (midline) hit the stationary object at the bottom of the fall. Male (*n* = 2)^392,413^ and female (*n* = 1)^413^ adult rats were used with these models. Mortality and NSS were not reported with this model. Righting reflex 1–10 min was reported in two publications.^392,413^

A pendulum injury model is another older model used to induce mTBI.^238,394,398^ In all of these publications, the animals were anesthetized. One publication did not fix the head^394^ whereas the other two used a bite bar to secure the head.^238,398^ All injuries were to the scalp of rats, and only adult males were used. No mortality rates were reported, and when a righting reflex was reported, the latency was 1–10 min.^394^ NSS was reported with deficits after mTBI in one publication.^398^

Two models were developed that we defined as projectile models. The Walter Reed Army Institute of Research developed a model called the WRAIR Projectile Concussive Impact model.^387,395^ In this model, anesthetized animals are placed on their back on a movable platform above a heating unit. Within the heating unit, they place a microcentrifuge tube filled with dry ice with a secure cap, causing the ice to sublimate, and the pressure causes the cap to be shot off and hit the helmet from below.^387^ The head is not fixed, but the body of the animal is held in place with an elastic band. This model used adult, male rats that were anesthetized and impacted laterally on the intact skull with a helmet. One other model from 1984 used a projectile to induce mTBI in adult male rats.^405^ In this model, a dart was shot at midline of the exposed scalp of an anesthetized animal. Mortality rates and the NSS were not reported for these models. Only one publication reported a righting reflex latency of 1–10 min.^395^

MicroTBI is a unique surgical technique used to cause increased pressure to the surface of the brain.^159,227,397^ This technique first surgically thins the skull of the mice and then applies pressure for a certain period to cause a brain injury. The head is fixed within a stereotactic frame through the injury procedure. Animals are anesthetized, the injury is caused laterally on the left somatosensory cortex of adult male mice. Mortality, righting reflex, and the NSS were not reported with this model.

The Maryland model of mTBI uses the energy of steel ball rolling down a 2.1-m track, hitting a coupling device that causes the impact to be centered on the malar processes to impact the front of the head.^391,393^ Rats are anesthetized and taped in place to reduce movement. The skull is not exposed in this model; however, surgery is done in order to expose the malar processes in which the injury device is positioned. The head is fixed in place within the device and is not free to move after the impact. Both publications with this model used adult male rats. One publication reported a mortality rate of 0–5%,^393^ and the other reported no deficit in the NSS.^391^ Righting reflex was not reported for this model.

Finally, one model from 1997 used a rubber reflex hammer that is fixed to a pivot for injury production.^390^ This injury was induced onto the intact scalp of adult of anesthetized adult male rats. Mortality, righting reflex, and the NSS were not reported with this model.

#### “Other” model: model and animal-specific common data elements

**FIG. 8.**
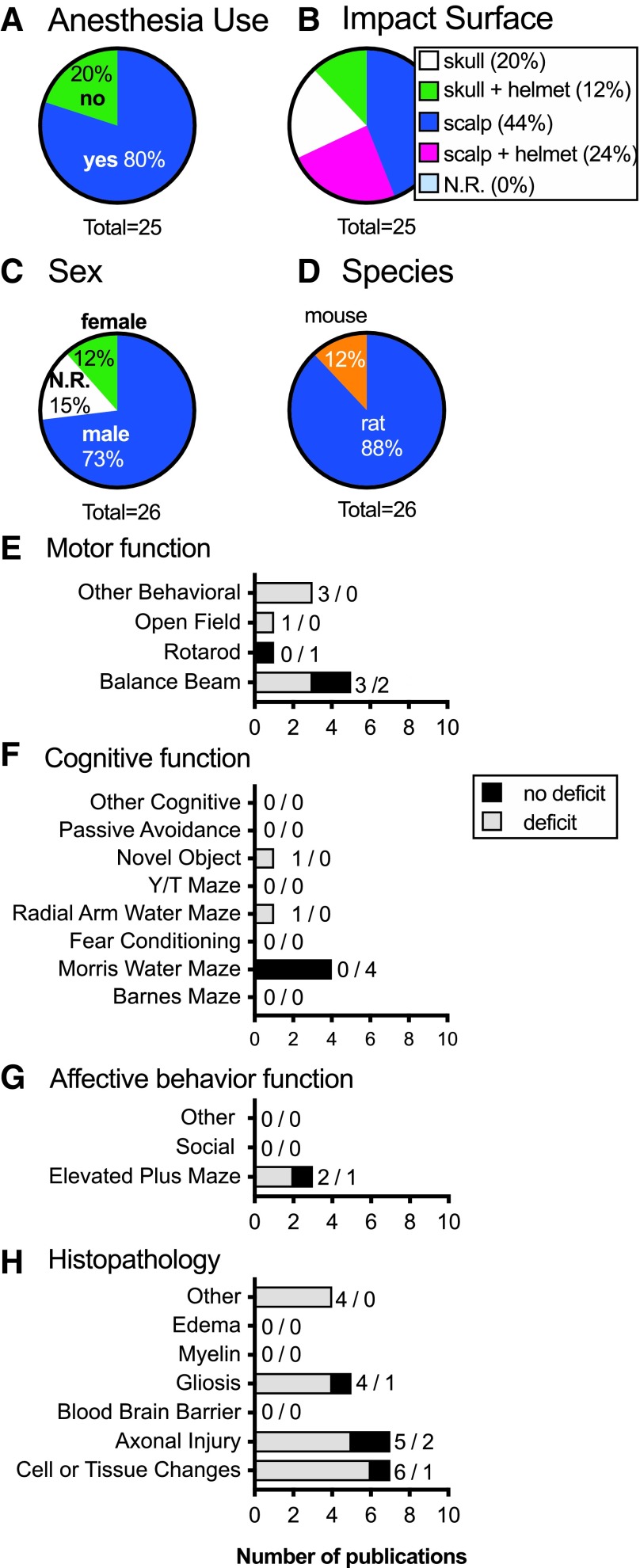
“Other” models common data elements. Most publications used anesthesia (**A**). Impact locations varied between models (**B**). However, most publications used males (**C**), and rats (**D**). Motor function (**E**), cognitive function (**F**), affective behaviors (**G**), and histological changes (**H**) were recorded for all “other” models. Numbers indicate the number of studies with / without deficits. Total numbers are greater than the 25 total publications in (C) and (D) because several publications reported using more than one sex and species. N.R., not reported. Color image is available online.

In this group as a whole, anesthesia was used in 80% of publications (Fig. 8A; SA 3.2), and the head was fixed in 84% of publications (SA 3.3). Different impact locations were used; 24% scalp with a helmet; 12% on the skull with a helmet; 44% to the intact scalp; and 20% to the skull (Fig. 8B; SA 3.4–3.7). Rats were used in 88% of publications (Fig. 8C; SA 3.8). A majority of the publications (76%) used male animals, regardless of species (Fig. 8D; SA 3.9). Of the publications that used females, one used both males and females and they did look at sex differences in their outcomes.^413^ All of these publications used adult animals.

#### “Other” model: injury-induced functional and histopathological changes

Behavioral outcomes were collected for these models as well. Motor behaviors were reported in 28% of these “other” models. The balance beam test commonly found deficits (3/2) (*n* = deficit/no deficit), and in one publication in the open field test (Fig. 8E; SA3.10). Rotarod was tested in one publication, and no deficits were found (SA 3.11). Other behavioral tests conducted included incline plane (1/0), gait analysis (1/0), and vision changes (1/0; *n* = deficit/no deficit; SA 3.12). Cognitive outcomes were reported in 24% of these publications (Fig. 8F). Testing in the main measurements was only observed for the Morris water maze (0/4), the radial arm maze (1/0), and novel object recognition (1/0; *n* = deficit/no deficit; SA 3.13). No other cognitive testing was reported in any of these “other” models. Of the common affective behavior tests, only the elevated plus maze was reported (2/1; *n* = deficit/no deficit; Fig. 8G; SA 3.14).

Histology, much more common in this category as compared to behavioral testing, was reported in 52% of publications (Fig. 8H). Cell and tissue changes were found in six publications, with one publication finding no deficits (SA 3.15). Similarly, axonal injury histological marker deficits were found in five publications, with two finding no deficits (SA 3.16). Gliosis activation was tested in five publications, and four found deficits while one found none (SA 3.17). Other histology included complement activation (1/0), doublecortin (1/0), brain-derived neurotrophic factor (1/0), and aquaporin 4 (1/0; *n* = deficit/no deficit; SA 3.18) (see online supplementary material).

## Discussion

In this systematic review, we identified and screened 1890 publications and summarized 402 original scientific reports of rodent CHI models of mTBI. We provide a summary of the common data elements used in these mTBI models to help define the current state of the field of rodent pre-clinical mTBI models. Identifying the common data elements is essential for big data efforts, such as the Federal Interagency Traumatic Brain Injury Research (FITBIR) informatics system (fitbir.nih.gov). We believe that our review will provide a framework for future research by summarizing the similarities and differences between models of CHI for mTBI, in both the ways the models were conducted and major classes of outcomes that were measured. Moreover, this review highlights the need for a consensus agreement on the best practices for pre-clinical models of mTBI CHI to both help new investigators move into the field and provide some degree of standardization of the common data elements used across laboratories. In addition to our review of the common data elements and outcome variables, excellent past reviews have discussed the TBI models of all severity types and comprehensively looked specifically at strengths and weaknesses of the different TBI models. We recommend that the interested reader consider the following reviews.^5,414–422^

In comparison to pre-clinical models of moderate-to-severe TBI, the number of publications attempting to model mild TBI is much smaller in number. At the same time, there is currently increased excitement in the field for developing mTBI models, in part because of the alarming number of people that suffer a mTBI, and the appreciation that an mTBI can have a lasting negative impact on the health of the brain. As new models are developed and characterized, they will add significantly to our understanding of the natural history of mTBI and how different biomechanical forces alter that history. Our review will help place emerging data into a historical context. By comparing how the values of injury model common data elements vary, and thus result in similar or disparate outcomes, can help to identify the most important aspects of the model that are leading to increased variability or are critical for causing a particular histopathological or functional outcome.

Before the early 1980s, there were no commercially available experimental models of TBI, which accounts for many of the “other”’ models found from our search criteria (Fig. 2D). Further, during this time period, many researchers were using cats and dogs to study TBI^423^ and therefore would have been excluded from this review that pertains specifically to rodent models: a potential limitation of our review.

In 1989, a model was produced and marketed by General Motors Research Laboratory that changed the trajectory of TBI research.^414^ The production of this piston-driven model was the early precursor to the CCI model—described initially in ferrets and later adapted for use in rodents.^424^ We mention the General Motors model here because of its contribution to commercially available models. In 2007, the Impact One^™^ was introduced to the market as a cheaper, user-friendly, and more space economical version of a piston impactor, which again changed the trajectory of TBI research.^425^ Introduced by Marmarou and colleagues in 1994,^179^ the weight drop model was another major advancement in the field of CHI, given that the device itself was easily producible in individual labs and continues to be widely used in the TBI field.

The presence of many different models can be a strength given that the differences between each one provides an opportunity to study varying mechanics of injury. We recognized that common data elements, which we currently view as important, were left out of many publications. For example, weight drop publications often did not include all details about the foam used to support the animals. It is known that specific foams can be used a finite number of times with a specific amount of time between use before the mechanical properties change.^426^ Thus, the understanding of the mechanical properties of the foam are important to report for future studies to ensure reproducibility. Similarly, within the piston-driven models, not all articles reported the impactor tip shape. In the CCI model, use of a flat or a rounded impact tip was found to change the progression and severity of the injury^427^; thus, the shape of the impact tip is important to know for understanding the injury produced. Although the surface of the brain is not exposed for the injury models in this review, it is reasonable to assume that the same principle will apply to CHIs. We mention these examples not to suggest that publications we reviewed which failed to report foam properties or impactor tip shape are flawed studies. For many of these studies, it was unknown at the time that foam properties or impactor tip shape were important. Nevertheless, having well-defined reporting standards for future studies will improve our ability to make cross-study comparisons. Along with ongoing efforts such as FITBIR, which will define national common data element reporting standards, this review will also help in providing context to the standards.

### Future directions

A striking finding from this work is the general lack of female animals used in mTBI models. This creates a huge gap in the understanding of brain injury responses specific to females. In 2000, a meta-analysis was conducted on clinical TBI studies which indicated poorer outcomes for females after a TBI.^428^ Differences between sex hold true also with pre-clinical animal model TBI.^429^ Many studies are not statistically powered to analyze the data by sex, given that the effect of sex may not be a specific aspect of their hypothesis. Nevertheless, for studies that include both male and female animals, future big data efforts of merging studies to look for effects of sex will only be possible if more researchers include both sexes in their studies.

Another major gap identified by this systematic review is the lack of both models of pediatric and geriatric mTBI. The CDC indicates that the rates of mTBI are at least several times higher in children compared to adults.^1^ Further, higher rates of mTBI are noted in the geriatric population (>65 years of age) compared to adults.^3^ Future studies addressing similarities and dissimilarities between adult animals and animals at the opposite ends of the aging spectrum will be useful to understand what aspects, if any, of the adult preclinical mTBI models are relevant to these two most at-risk patient populations.

Of the various dependent variables measured and collected in our review, we also collected the time point at which each publication looked at their variable. From this information, we found that very few of the 403 publications looked further than 1 month after mTBI. With the CDC reporting that around 5.3 million individuals live with a permanent TBI-related disability, the lack of long-term studies in the literature is a major gap that needs to be filled in the field.^1^

### Limitations

Not only within the context of this review, but in the TBI field as a whole, the definition of mTBI can vary from study to study. Most often, mTBI and concussion are used interchangeably. Experts have attempted to establish universal guidelines, but they have only been able to agree on the fact that mTBI represents a “change in brain activity from a traumatic force.”^3^ Changes in brain function can be so subtle that they may be difficult to detect, as observed in some of the publications included in this review in which no deficits in behavior or histological measures could be found after a traumatic blow to the head of a rodent. While we attempted to only include publications that were mild, we relied on the author's criteria for a mild injury, which can be rather varied.

The guideline for systematic reviews published by PRISMA includes a section to address bias within and between the articles used for the review.^7^ We did not address the quality of the publications included in the final analysis. The aim of this review was to compile a list of closed head mTBI rodent models and simply provide which tests have been done within those publications. Future work may use the models found from this review and retrieve information from the publications about the time course of pathology or behavior changes noted and compare across studies.

When outcome measures were recorded from the 402 publications, certain tests, such as the open field and the passive avoidance test, were kept within one category, either motor or cognitive respectively. These tests have multiple aspects, however, that can measure different parts of rather complex behaviors. Further investigation to determine which factor was the main purpose of the test for each article's experimental design is warranted.

## Conclusion

We have compiled over 400 articles all using a closed head mild injury model to study TBI in rodents. Not only are all of these articles now in one place, but further information about the common data elements of the injury protocol, the animals used, and the outcomes measured have also been compiled and summarized. We believe that this review will be helpful for understanding where the closed head mTBI field has been, where gaps are present that need to be addressed, and what the future holds for the field.

## Supplementary Material

Supplemental data
