# Supplementary material for: A Systematic Review of Closed Head Injury Models of Mild Traumatic Brain Injury in Mice and Rats
Source: J Neurotrauma. 2019 May 22;36(11):1683–706. doi: 10.1089/neu.2018.6127 (PMC6555186; doi:10.1089/neu.2018.6127)
Supplement: Supplemental data [file Supp_App.docx]

| Supplementary appendix: Original Publication References | | | |
| --- | --- | --- | --- |
| Category | **Pubmed ID or DOI (when no PubMed ID is available)** | | |
| 0.1 dependent variable time point | **Up to 1 day:** 24220698, 23558510, 10674756, 17000527, WOS:000183356100015, 17612919, 25886256, 25879458, 21740439, 8929504, 8738249, 25168788, 22860603, 24013757, 14702209, 23591210, WOS:000390627300006, 10486200, 15168230, 21988140, 10.5336/medsci.2009-11356, 19998488, 19018450, 17288002, 15254773, 24810171, 25438952, 16004578, 11226099, 9416298, 10972243, 12657997, 14521991, 9356065, 8683262, 23122881, 20850781, WOS:A1997WL11600016, 27365176, 14577862, 9361992, 26806371, 16758355, 20617879, 9204117, 458474, 19215660, WOS:000377289200002, 17033555, 21325251, 21466884, 21274455, 21394044, 23667624, 11597656, 16456449, 12589996, 9779197, 28070456, 7976618, 2444951, 10494671, 26502232, 9688127, 10447072, 17610350, 24756867, 27521753, 25957560, 23743262, 18590543, 10101254, 9130293, 23960212, 27006282, 10936684, 24387843, 11782990, 23262351, 19605168, 8283269, 11345518, 16369137, 25081505, 11744229, 26972829, 25555930, 25548960, 8622802, 7629865, 874549, 874550, 22149927, 7968229, 8412497, 5170668, 16689671, 11435946, 27744073, 28461271, 15972243, 15729296, 19691993, 11320592, 10595822, 22503903, 27167127, 18447626, 14659826, 9779153, 25106629, 10.1001/archneurpsyc.1941.02280130011001, 8121612, 17289399, 9559767, 9785592, 19812968, 18077582, 4070440, 3016201, 3416806, 6093755, 19723705, 26157981, 2477500, 1786258, 8014208, 9042110, 8621742, 1295574, 7773695, 23178198, 26458361, 10899295, 10208272, 9112087, 9728831, 9620509, 16689670, 9062682, 9421457, 12225654, 23884669, 10454350, 10547099, WOS:000085570200025, 16671445, 17264861, 23937270, 23198799, 25873133, 10391365, 23622728, 24088994, 9433909, 26939762, 24938400, 20493841, 20951803 | | |
|  | **Up to 1 week:** 19958766, 11686497, 22569191, 22450152, 27602324, 26275125, 16822489, 9119900, 14597558, 19484631, 9462472, 17188268, 26124685, 10527147, 5091699, 22424792, 21299360, 15289185, 25872478, 23608674, 18440507, 29957302, 19429018, 10873515, 29648487, 11101205 , 15850087, 28194658, 29746557, 27615406, 17711400, 19713954, 8283270, 29463176, 27363926, 18407501, 28383046, 29367701, 10797559, 26541248, 18159994, 7477676, 27657499, 25290011, 26081154, 9609302, 19660237, 28038986, 15036604, 16084512, 27138134, 28552947, 10082192, 11780864, 12707777, 19317611, 26537880, 17626731, 26605126, 9203544, 10.4274/haseki.3446, 27278330, 27923162, 20940727, 19929375, 29344708, 16455070, 16545803, 16842619, 17474994, 26474659, 28284950, 9797032, 25394735, 22498027, 28214984, 11147383, 11336441, 17711399, 22498103, 22652305, 16459044, 24231261, 25270295, 18240300, 28558476, 17600512, 11526986, 24691765, 11706345, 29665077, 16864909, 23984869, 23808389, 27188531, 20138993, 25576803, 28988852, 18640839, 15815471, 8317722, 18260794, 23040263, 3164097, 8411215, 7976614, 19688182, 24239694, 27779341, 26216663, 21770756, 21904255, 10698075, 10996218, 19133139, 25344352, 23327111, 9421456, 9062683, 9313933, 18651249, 11450053, 19468925, 26905805, 23059457, 24124534, 15987552, 24861442, 16960300, 23354401, 28685977, 22335783, 7496806, 22120305, 25450468, 27155455, 28659494, 7652031, 21871613, 21979171 | | |
|  | **Up to 1 month:** 18240300, 24640955, 27285176, 28690173, 20001584, 9384402, 27899434, 26414556, 23184513, 9284075, 20486807, 21505478, 28288867, 26528156, 25124230, 21120456, 17270455, 11083921, 9416283, 23294038, 26448536, 26709201, 28514188, 17828037, 26912636, 25241288, 27988361, 12225655, 10636620, 15002855, 20812776, 25982185, 10965994 , 26039099, 25770855, 27547849, 15698624, 25721934, 29499284, 18702633, WOS:000228280200023, 7723065, 28761433, 26484783, 25698949, 24283269, 28274861, 8909984, 9192383, 27498246, 24312187, 11223919, 23443171, 22837752, 25025304, 21219958, 26529240, 20621186, 25379886, 15902197, 27357503, 27373531, 17188500, 27188340, 29330386, 27230970, 26868732, 20507613, 17521631, 27735217, 28780369, 17351125, 22034986, WOS:000231843700015, 16042992, 22129535 | | |
|  | **Over 1 month:** 26258958, 7475922, 27072754, 28855139, 28321191, 27845195, 10.5812/archneurosci.38039, 25668562, 16156715, 17669633, 11931348, 24158195, 22314279, 21910642, 22173016, 21561314, 24957202, 26756169, 25904805, 26153729, 29739276, 12732240, 16356639, 21499325 | | |
|  | **Over 1 year:** 27540748, 27656033 | | |
|  |  | | |
|  |  | | |
| Weight Drop | | | |
| 1.1 Rats | 10674756, 17000527, 17612919, 8929504, 8738249, 22860603, 14702209, 23591210, 10486200, 15168230, 10.5336/medsci.2009-11356, 17288002, 15254773, 11226099, 9416298, 10972243, 12657997, 9356065, 8683262, WOS:A1997WL11600016, 27365176, 16758355, 9204117, 19215660, 17033555, 11597656, 16456449, 12589996, 9779197, 28070456, 7976618, 10494671, 9688127, 10447072, 27521753, 10936684, 23262351, 8283269, 11345518, 16369137, 11744229, 26972829, 25555930, 25548960, 7629865, 7968229, 8412497, 5170668, 11686497, 22450152, 27602324, 26275125, 9119900, 19484631, 9462472, 26124685, 10527147, 25872478, 23608674, 29957302, 19429018, 10873515, 11101205 , 28194658, 8283270, 27363926, 18407501, 18159994, 7477676, 25290011, 9609302, 19660237, 16084512, 28552947, 12707777, 17626731, 26605126, 9203544, 10.4274/haseki.3446, 29344708, 11147383, 11336441, 17711399, 22498103, 16459044, 24231261, 25270295, 18240300, 28558476, 9384402, 27899434, 26414556, 9416283, 23294038, 26448536, 17828037, 27988361, 12225655, 10636620, 15002855, 28761433, 26484783, 25698949, 8909984, 9192383, 26258958, 7475922, 27072754, 28855139, 12777900, 20617879, 23667624, 3229660, 7583233,28461271, 15972243, 15729296, 19691993, 11320592, 10595822, 22503903, 27167127, 11526986, 24691765, 11706345, 27498246, 24312187, 14659826, 9779153, 17289399, 9559767, 23984869, 25576803, 8317722, 19812968, 18077582, 4070440, 2477500, 1786258, 8014208, 9042110, 1295574, 7773695, 26458361, 10208272, 9112087, 9728831, 9620509, 16689670, 23884669, 10454350, 16671445, 23937270, 3164097, 8411215, 7976614, 19688182, 24239694, 27779341, 26216663, 10996218, 23327111, 9421456, 19468925, 26905805, 15987552, 24861442, 25379886, 27357503, 17188500, 29330386, 27230970, 20507613, 21910642, 26756169, 26868732, 10659149, 10391365, 16960300, 23354401, 7496806, 27735217, 23046422, 22120305, 27155455, 22034986, 16099948, 20493841, 7652031, 21979171, 22129535, 9433909 | | |
| 1.2 Mice | 24220698, 23558510, 25879458, 21740439, 19958766, 22569191, 27285176, 20001584, 25168788, 24013757, 19998488, 19018450, 24810171, 25438952, 16004578, 14521991, 23122881, 20850781, 21325251, 21466884, 21274455, 21394044, 26502232, 23743262, 10101254, 9130293, 23960212, 27006282, 24387843, 19605168, 14597558, 17188268, 22424792, 15289185, 29746557, 27615406, 19713954, 29463176, 29367701, 10797559, 26081154, 15036604, 10082192, 11780864, 19317611, 26537880, 27278330, 27923162, 16545803, 16842619, 17474994, 26474659, 28284950, 9797032, 25394735, 22498027, 28214984, 28690173, 9284075, 21505478, 28288867, 26528156, 25124230, 21120456, 17270455, 11083921, 28514188, 26912636, 10965994 , 26039099, 25770855, 27547849, 25721934, 29499284, 28321191, 10.5812/archneurosci.38039, 23184513, 28383046, 14577862, 27266706, 20940727, 25081505, 874550, 11435946, 27744073, 22652305, 28274861, 16156715, 17669633, 25106629, 17600512, 29665077, 16864909, 28988852, 11223919, 21219958, 20621186, 8121612, 9785592, 26157981, 8621742, 23178198, 9062682, 9421457, 12225654, 10547099, 25873133, 23622728, 24088994, 26939762, 20951803, 15815471, 18260794, 23040263, 19688182, 21770756, 21904255, 10698075, 19133139, 9062683, 9313933, 18651249, 11450053, 23059457, 24124534, 28685977, 22335783, 28659494, 21871613, 15902197, 27188340, 17351125, 16042992, 22314279, 22173016, 21561314, 24957202, 12732240, 16356639, 21499325, 16099948, 9406936, 25450468, 24938400 | | |
| 1.3 Both Rats and Mice | 19688182, 16099948 | | |
| 1.4 Sex | **Males:** 24220698, 23558510, 10674756, 17000527, 17612919, 25879458, 21740439, 8929504, 8738249, 25168788, 22860603, 24013757, 14702209, 23591210, 15168230, 10.5336/medsci.2009-11356, 19998488, 19018450, 17288002, 15254773, 24810171, 25438952, 16004578, 9416298, 10972243, 12657997, 14521991, 9356065, 8683262, 23122881, 20850781, 16758355, 9204117, 19215660, 21325251, 21466884, 21274455, 21394044, 16456449, 9779197, 28070456, 7976618, 10494671, 26502232, 9688127, 10447072, 27521753, 23743262, 9130293, 19958766, 11686497, 22569191, 22450152, 27602324, 26275125, 9119900, 14597558, 9462472, 17188268, 26124685, 22424792, 15289185, 23608674, 29957302, 19429018, 10873515, 11101205 , 28194658, 29746557, 27615406, 19713954, 8283270, 27363926, 18407501, 29367701, 10797559, 18159994, 7477676, 25290011, 26081154, 19660237, 15036604, 16084512, 28552947, 10082192, 11780864, 12707777, 19317611, 26537880, 17626731, 26605126, 9203544, 27278330, 27923162, 29344708, 16545803, 16842619, 17474994, 26474659, 28284950, 9797032, 25394735, 22498027, 28214984, 24640955, 27285176, 28690173, 20001584, 27899434, 26414556, 9284075, 21505478, 28288867, 25124230, 21120456, 17270455, 11083921, 23294038, 28514188, 17828037, 27988361, 12225655, 26258958, 7475922, 27072754, 28855139, 28321191, 20617879, 23667624, 23184513, 28383046, 14577862, 27266706, 20940727  10101254, 23960212, 10936684, 24387843, 19605168, 11345518, 16369137, 11744229, 7629865, 874550, 7968229, 8412497, 5170668, 11435946, 27744073, 28461271, 15972243, 19691993, 11320592, 10595822, 22503903, 27167127, 14659826, 9779153, 11147383, 11336441, 17711399, 22498103, 22652305, 16459044, 18240300, 28558476, 17600512, 11526986, 11706345, 29665077, 16864909, 10965994 , 26039099, 25770855, 27547849, 25721934, 28274861, 8909984, 9192383, 27498246, 11223919, 21219958, 16156715, 17669633, 3229660, 25106629, 17289399, 9559767, 9785592, 19812968, 18077582, 4070440, 2477500, 1786258, 8014208, 9042110, 8621742, 1295574, 7773695, 23178198, 26458361, 10208272, 9112087, 9728831, 9062682, 9421457, 12225654, 10454350, 16671445, 23937270, 25873133, 10391365, 23622728, 24088994, 26939762, 20493841, 20951803, 28988852, 8317722, 18260794, 23040263, 3164097, 8411215, 7976614, 24239694, 27779341, 26216663, 21770756, 21904255, 10698075, 10996218, 19133139, 23327111, 9421456, 9062683, 9313933, 18651249, 11450053, 23059457, 15987552, 24861442, 16960300, 23354401, 22335783, 7496806, 27155455, 28659494, 7652031, 21979171, 20621186, 15902197, 27357503, 17188500, 27188340, 29330386, 20507613, 17351125, 22034986, 16042992, 22129535, 22314279, 21910642, 22173016, 21561314, 24957202, 26756169, 12732240, 16356639, 21499325, 10659149, 22120305, 16099948, 9433909, 9406936, 25450468, 24938400 | | |
|  | **Females:** 27365176, 11597656, 27006282, 23262351, 25081505, 26972829, 25555930, 25548960, 15729296, 29463176, 24231261, 15815471, 19688182, 19468925, 26905805, 28685977, 26528156, 26448536, 26912636, 15002855, 29499284, 28761433, 26484783, 25698949, 27230970, 27735217, 10.5812/archneurosci.38039, 7583233, 26868732 | | |
| 1.5 Both Males and Females | **Analyzed by sex:** 25555930, 25548960, 24231261, 25270295, 26448536, 26484783, 25698949, 24283269 | | |
|  | **Did not analyze by sex:** 27365176, 25081505, 25555930, 25548960, 29463176, 24231261, 15815471, 19688182, 19468925, 28685977, 26448536, 26912636, 29499284, 26484783, 25698949, 10.5812/archneurosci.38039, 7583233 | | |
| 1.6 Young | **Rats:** 27365176, 25555930, 25548960, 11686497, 18159994, 24231261, 19688182, 19468925, 16960300, 26448536, 26484783, 7583233  **Mice:** 15815471, 19688182 | | |
| 1.7 Adults | 24220698, 23558510, 10674756, 17000527, 17612919, 25879458, 21740439, 8929504, 8738249, 25168788, 22860603, 24013757, 14702209, 23591210, 10486200, 15168230, 10.5336/medsci.2009-11356, 19998488, 19018450, 17288002, 15254773, 24810171, 25438952, 16004578, 11226099, 9416298, 10972243, 12657997, 14521991, 9356065, 8683262, 23122881, 20850781, WOS:A1997WL11600016, 16758355, 9204117, 19215660, 17033555, 21325251, 21466884, 21274455, 21394044, 11597656, 16456449, 12589996, 9779197, 28070456, 7976618, 10494671, 26502232, 9688127, 10447072, 27521753, 23743262, 10101254, 9130293, 23960212, 27006282, 10936684, 24387843, 23262351, 19605168, 8283269, 11345518, 16369137, 11744229, 26972829, 7629865, 874550, 7968229, 8412497, 11435946, 27744073, 28461271, 15972243, 15729296, 19691993, 11320592, 10595822, 22503903, 27167127, 14659826, 9779153, 25106629, 8121612, 17289399, 9559767, 9785592, 19812968, 18077582, 4070440, 26157981, 2477500, 8014208, 9042110, 8621742, 1295574, 7773695, 23178198, 26458361, 10208272, 9112087, 9728831, 9620509, 16689670, 9062682, 9421457, 12225654, 23884669, 10454350, 10547099, 16671445, 23937270, 25873133, 10391365, 23622728, 24088994, 26939762, 20493841, 20951803, 19958766, 22569191, 22450152, 27602324, 26275125, 9119900, 14597558, 19484631, 9462472, 17188268, 26124685, 10527147, 22424792, 15289185, 25872478, 23608674, 29957302, 19429018, 10873515, 11101205 , 28194658, 29746557, 27615406, 19713954, 8283270, 29463176, 27363926, 18407501, 29367701, 10797559, 7477676, 25290011, 26081154, 9609302, 19660237, 15036604, 16084512, 28552947, 10082192, 11780864, 12707777, 19317611, 26537880, 17626731, 26605126, 9203544, 27278330, 27923162, 29344708, 16545803, 16842619, 17474994, 26474659, 28284950, 9797032, 25394735, 22498027, 28214984, 11147383, 11336441, 17711399, 22498103, 22652305, 16459044, 25270295, 18240300, 28558476, 17600512, 11526986, 24691765, 11706345, 29665077, 16864909, 23984869, 28988852, 8317722, 18260794, 23040263, 3164097, 8411215, 7976614, 24239694, 27779341, 26216663, 21770756, 21904255, 10698075, 10996218, 19133139, 23327111, 9421456, 9062683, 9313933, 18651249, 11450053, 26905805, 23059457, 24124534, 15987552, 24861442, 23354401, 28685977, 22335783, 7496806, 27155455, 28659494, 7652031, 21871613, 21979171, 24640955, 27285176, 28690173, 20001584, 9384402, 27899434, 26414556, 9284075, 21505478, 28288867, 26528156, 25124230, 21120456, 17270455, 11083921, 9416283, 23294038, 28514188, 17828037, 26912636, 27988361, 12225655, 10636620, 15002855, 10965994 , 26039099, 25770855, 27547849, 25721934, 29499284, 28761433, 25698949, 28274861, 8909984, 9192383, 27498246, 11223919, 21219958, 20621186, 25379886, 15902197, 27357503, 17188500, 27188340, 29330386, 27230970, 20507613, 27735217, 17351125, 22034986, 16042992, 22129535, 26258958, 7475922, 27072754, 28855139, 28321191, 10.5812/archneurosci.38039, 16156715, 17669633, 22314279, 21910642, 22173016, 21561314, 24957202, 26756169, 12732240, 16356639, 21499325, 12777900, 20617879, 23667624, 3229660, 24312187, 26868732, 10659149, 23046422, 22120305, 16099948, 9433909, 23184513, 28383046, 14577862, 27266706, 20940727, 9406936, 25450468, 24938400 | | |
| 1.8 Aged | 11744229, 5170668 | | |
| 1.9 No Anesthesia | 28070456, 19605168, 9797032, 7652031, 15002855, 3229660 | | |
| 1.10 Skull Only | 17612919, 8738249, 15168230, 19018450, 24810171, 25438952, 16004578, 9416298, 10972243, 14521991, 8683262, 20850781, WOS:A1997WL11600016, 16758355, 17033555, 12589996, 10101254, 9130293, 24387843, 11345518, 16369137, 25081505, 11744229, 8412497, 27744073, 14659826, 9779153, 8121612, 9559767, 9785592, 19812968, 4070440, 2477500, 1786258, 8014208, 9042110, 1295574, 7773695, 9620509, 16689670, 19958766, 22569191, 26275125, 9119900, 14597558, 17188268, 10527147, 11101205 , 19713954, 29463176, 27363926, 18407501, 29367701, 10797559, 18159994, 26081154, 9609302, 16084512, 10082192, 11780864, 27278330, 27923162, 29344708, 16545803, 16842619, 17474994, 25394735, 22498027, 28214984, 11147383, 17711399, 22498103, 16459044, 18240300, 17600512, 24691765, 11706345, 29665077, 16864909, 25576803, 28988852, 15815471, 8317722, 18260794, 23040263, 3164097, 8411215, 7976614, 26216663, 10698075, 19133139, 11450053, 24640955, 26414556, 9284075, 21505478, 26528156, 17270455, 11083921, 23294038, 17828037, 26912636, 12225655, 10636620, 10965994 , 25721934, 29499284, 28274861, 11223919, 20621186, 25379886, 15902197, 17188500, 27188340, 29330386, 28321191, 10.5812/archneurosci.38039, 17669633, 22173016, 21561314, 12777900, 24312187, 23184513, 28383046, 27266706, 9406936  10547099, 25873133, 26939762, 28685977, 22335783, 27155455, 28659494, 20507613, 27735217, 17351125, 16042992, 24957202, 16099948, 20493841, 20951803, 21871613, 21979171, 22129535 | | |
| 1.11 Skull + Helmet | 10674756, 17000527, 8929504, 22860603, 14702209, 23591210, 10486200, 10.5336/medsci.2009-11356, 17288002, 15254773, 12657997, 9204117, 19215660, 11597656, 16456449, 9779197, 7976618, 10494671, 9688127, 10447072, 27521753, 10936684, 23262351, 8283269, 26972829, 7629865, 874550, 28461271, 15972243, 19691993, 11320592, 10595822, 22503903, 27167127, 17289399, 18077582, 26157981, 26458361, 10208272, 9112087, 23884669, 10454350, 23937270, 10391365, 11686497, 22450152, 27602324, 19484631, 9462472, 26124685, 22424792, 25872478, 23608674, 29957302, 19429018, 10873515, 8283270, 7477676, 25290011, 19660237, 28552947, 12707777, 17626731, 26605126, 9203544, 11336441, 28558476, 11526986, 23984869, 19688182, 24239694, 10996218, 23327111, 9421456, 19468925, 15987552, 24861442, 23354401, 9384402, 9416283, 27988361, 27498246, 27357503, 22034986, 26258958, 7475922, 27072754, 28855139, 26756169, 20617879, 23667624, 10659149, 23046422, 22120305, 9433909, 25450468 | | |
| 1.12 Scalp Only | 24220698, 23558510, 25879458, 21740439, 25168788, 24013757, 19998488, 11226099, 9356065, 23122881, 27365176, 21325251, 21466884, 21274455, 21394044, 26502232, 23743262, 23960212, 27006282, 19605168, 15289185, 29746557, 15036604, 19317611, 26537880, 26474659, 28284950, 9797032, 27285176, 28690173, 20001584, 27899434, 28288867, 25124230, 21120456, 26448536, 28514188, 15002855, 26039099, 25770855, 27547849, 14577862, 20940727,25555930, 25548960, 7968229, 5170668, 11435946, 15729296, 25106629, 8621742, 23178198, 9728831, 9062682, 9421457, 12225654, 23622728, 24088994, 22652305, 24231261, 25270295, 27779341, 21770756, 21904255, 9062683, 9313933, 18651249, 23059457, 24124534, 16960300, 7496806, 7652031, 26484783, 25698949, 8909984, 9192383, 21219958, 16156715, 22314279, 21910642, 12732240, 16356639, 21499325, 3229660, 7583233, 24938400 | | |
| 1.13 Scalp + Helmet | 28070456, 16671445, 28194658, 27615406, 26905805, 27230970, 26868732 | | |
| 1.14 FIxed in ear bars | 24220698, 17612919, 15168230, 9416298, 10972243, WOS:A1997WL11600016, 17033555, 12589996, 24387843, 16369137, 25081505, 14659826, 9779153, 25873133, 22569191, 10527147, 9609302, 11780864, 11147383, 24691765, 19688182, 28685977, 7496806, 7652031, 27988361, 12225655, 10636620, 3229660, 24312187 | | |
| 1.15 fixed between blocks | 16084512, 22498103, 26216663, 23294038, 17828037 | | |
| 1.16 held in the hand of the experimenter | 23122881, 19605168, 19958766, 19713954, 27278330, 9797032, 17711399, 18260794, 23040263, 28274861, 11223919, 15902197, 21499325, 27266706 | | |
| 1.17 head on foam pad | 24220698, 23558510, 10674756, 25879458, 21740439, 25168788, 10486200, 10.5336/medsci.2009-11356, 19998488, 12657997, 9204117, 19215660, 11597656, 16456449, 10494671, 9688127, 10447072, 27521753, 10936684, 23262351, 19605168, 8283269, 26972829, 7629865, 874550, 7968229, 5170668, 28461271, 15972243, 27167127, 25106629, 17289399, 18077582, 8621742, 26458361, 10208272, 9112087, 9728831, 12225654, 23884669, 10454350, 23937270, 10391365, 20951803, 11686497, 22450152, 19484631, 26124685, 22424792, 25872478, 29957302, 19429018, 28194658, 27615406, 8283270, 7477676, 25290011, 19660237, 28552947, 12707777, 26537880, 17626731, 26605126, 26474659, 28284950, 25394735, 11336441, 22652305, 28558476, 11706345, 23984869, 27779341, 10996218, 23327111, 9421456, 19468925, 26905805, 23059457, 24861442, 23354401, 27285176, 28690173, 20001584, 27899434, 25124230, 28514188, 27988361, 8909984, 9192383, 27498246, 21219958, 27357503, 27230970, 22034986, 26258958, 7475922, 27072754, 28855139, 16156715, 21910642, 26756169, 16356639, 21499325, 20617879, 23667624, 26868732, 10659149, 23046422, 22120305, 9433909, 25450468 | | |
| 1.18 Head on foil | 27365176, 25555930, 25548960, 24231261, 25270295, 16960300, 26448536, 26484783, 25698949, 7583233 | | |
| 1.19 head on rubber | 9062682, 9421457, 9062683, 9313933, 18651249, 26912636 | | |
| 1.20 head on spring platform | 9416298, 10972243, 23743262, 16671445 | | |
| 1.21 Brass weight | 10674756, 25879458, 21740439, 8929504, 25168788, 15168230, 17288002, 15254773, 12657997, 23122881, 19215660, 21325251, 21466884, 21274455, 21394044, 11597656, 7976618, 19605168, 8283269, 7629865, 7968229, 5170668, 15729296, 19691993, 27167127, 25106629, 17289399, 19812968, 26157981, 26458361, 10208272, 9112087, 9728831, 9062682, 23884669, 10454350, 16671445, 25873133, 10391365, 11686497, 22450152, 19484631, 28194658, 18159994, 7477676, 25290011, 19660237, 28552947, 11780864, 12707777, 17626731, 26605126, 26474659, 28284950, 9797032, 22652305, 28558476, 11526986, 11706345, 25576803, 27779341, 10996218, 23327111, 9421456, 9062683, 19468925, 26905805, 23059457, 15987552, 24861442, 16960300, 23354401, 28685977, 7496806, 27285176, 28690173, 20001584, 9384402, 21505478, 28288867, 25124230, 21120456, 9416283, 28514188, 27988361, 15002855, 25721934, 8909984, 27498246, 20621186, 27357503, 27230970, 22034986, 26258958, 28855139, 28321191, 16156715, 21910642, 26756169, 20617879, 23667624, 3229660, 26868732, 10659149, 23046422, 9433909, 25450468 | | |
| 1.22 silicone tipped weight | 19018450, 16004578, 11226099, 9416298, 10972243, 14521991, 8683262, 17033555, 23743262, 16369137, 9559767, 1786258, 9042110, 16689670, 20493841, 22569191, 17188268, 10527147, 9609302, 29344708, 11147383, 24691765, 16864909, 8317722, 7652031, 21979171, 26414556, 17270455, 11083921, 10636620, 25379886, 27188340, 22129535, 12777900, 24312187, 27266706 | | |
| 1.23 Rat 450 g/100 cm | 11597656, 16456449, 9779197, 10494671, 28552947, 9203544, 28855139  8283269, 7629865, 28558476, 24239694, 15987552, 26756169 | | |
| 1.24 rat 450 g/200 cm | 8929504, 14702209, 10486200, 15254773, 22450152, 9384402, 26258958, 27072754  17288002, 12657997, 29957302, 19429018, 10873515, 7477676, 9416283, 20617879  9204117, 19215660, 7976618, 9688127, 10936684, 23262351, 8283269, 19691993, 10595822, 26458361, 10208272, 10454350, 10391365, 28552947, 17626731, 26605126, 11336441, 11526986, 23327111, 9421456, 26905805, 23354401, 27357503, 27230970, 22034986, 23667624, 26868732, 9433909 | | |
| 1.25 mice 80 cm | 25879458, 21740439, 25168788, 24013757, 19998488, 27285176, 28690173, 25124230, 19317611, 26537880, 26474659, 28284950, 25394735, 21120456, 28514188, 16156715, 25106629, 26157981, 12225654, 20951803, 22652305, 23059457, 24124534, 11223919, 21219958, 17669633, 12732240, 16356639, 21499325 | | |
| 1.26 mice Less than 2.5cm | 25879458, 21740439, 25168788, 24013757, 19998488, 19018450, 24810171, 25438952, 20850781, 23743262, 27744073, 25106629, 8121612, 26157981, 12225654, 23622728, 24088994, 20951803, 17188268, 29746557, 19713954, 29463176, 19317611, 26537880, 27278330, 27923162, 16545803, 16842619, 17474994, 26474659, 28284950, 25394735, 22498027, 28214984, 22652305, 28988852, 10698075, 19133139, 23059457, 24124534, 28685977, 28659494, 21871613, 27285176, 28690173, 26528156, 25124230, 21120456, 28514188, 29499284, 11223919, 21219958, 27188340, 10.5812/archneurosci.38039, 16156715, 17669633, 12732240, 16356639, 21499325, 23184513, 9406936 | | |
| 1.27 less than 2.5cm/333 g | 19018450, 23743262, 10101254, 9130293, 27744073, 8121612, 17188268, 29463176, 10797559, 10082192, 27923162, 16545803, 17474994, 22498027, 16864909, 22335783, 21871613, 10965994 , 29499284, 20621186, 27188340, 10.5812/archneurosci.38039, 22173016, 9406936 | | |
| 1.28 less than 2.5cm/lateral impact | 19018450, 24810171, 25438952, 23743262, 27744073, 8121612, 17188268, 29463176, 27278330, 27923162, 16545803, 17474994, 22498027, 28214984, 28988852, 10698075, 19133139, 28685977, 28659494, 21871613, 26528156, 27188340, 23184513, 9406936 | | |
| 1.29 less than 2.5 cm/ skull | 19018450, 24810171, 25438952, 20850781, 17188268, 19713954, 29463176, 27278330, 27923162, 16545803, 16842619, 17474994, 22498027, 28214984, 26528156, 10.5812/archneurosci.38039, 23184513  27744073, 8121612, 28988852, 10698075, 19133139, 28685977, 28659494, 21871613, 29499284, 27188340, 9406936 | | |
| 1.30 Rope attached to weight | 19484631, 19660237, 24231261, 19468925, 26756169 | | |
| 1.31 kimwipe or tin foil | 25555930, 25548960, 24938400, 20940727, 24231261, 25270295, 28685977, 26448536, 26709201, 26484783, 25698949, 24283269 | | |
| 1.32 NSS | **Deficits:** 24810171, 11226099, 9356065, 8683262, 23122881, WOS:A1997WL11600016, 19958766, 22569191, 27602324, 9119900, 19484631, 17188268, 10527147, 23608674, 11101205 , 19713954, 29367701, 25290011, 9609302, 19660237, 10082192, 9284075, 28288867, 26912636, 12777900, 23184513, 28383046, 27266706, 17033555, 12589996, 23743262, 23960212, 27006282, 25081505, 5170668, 8121612, 9559767, 9785592, 8014208, 7773695, 9112087, 9620509, 23884669, 25873133, 20493841, 27278330, 27923162, 29344708, 16545803, 16842619, 28214984, 11147383, 17711399, 24691765, 11706345, 29665077, 8317722, 18260794, 3164097, 7976614, 21770756, 19133139, 11450053, 28685977, 22335783, 27155455, 28659494, 21871613, 12225655, 10636620, 26039099, 25770855, 27547849, 25721934, 27498246, 20621186, 25379886, 15902197, 17188500, 27188340, 29330386, 17351125, 22129535, 22173016, 24957202, 3229660, 24312187, 23046422, 16099948, 9433909  **No deficits:** 24387843, 10547099, 20951803, 14597558, 17600512, 26414556, 28514188, 16042992, 16156715, 17669633, 21561314, 12732240, 21499325, 25450468 | | |
| 1.33 righting reflex | 15168230, 20850781, 19605168, 25555930, 25548960, 7629865, 7968229, 5170668, 9062682, 9421457, 15289185, 29746557, 27363926, 7477676, 26605126, 9797032, 24231261, 11706345, 19688182, 9062683, 27899434, 17828037, 25698949, 9192383, 27230970, 3229660, 7583233, 26868732, 10659149, 23184513, 20940727, 24938400 | | |
| 1.34 Low Mortality (0-5%) | 24220698, 17000527, 15168230, 10972243, 9356065, 23743262, 7629865, 8412497, 15729296, 9779153, 26458361, 22450152, 10873515, 28194658, 18159994, 7477676, 9797032, 22652305, 19688182, 9062683, 28659494, 26414556, 17828037, 9192383, 7475922, 16156715, 21561314, 12777900, 26868732, 10659149, 24938400 | | |
| 1.35 moderate mortality (5-30%) | 24810171, 11226099, 8683262, 23122881, 19215660, 21394044, 23960212, 27006282, 16369137, 11435946, 9559767, 23178198, 9062682, 23622728, 11101205 , 29746557, 19713954, 29463176, 25290011, 9609302, 28552947, 29344708, 24691765, 23984869, 8317722, 21770756, 21904255, 22335783, 9384402, 28288867, 10636620, 26039099, 25770855, 27547849, 25721934, 27188340, 22034986, 16042992, 22314279, 14577862, 20940727, 25450468 | | |
| 1.36 high mortality (30%+) | 17288002, 9416298, 8283269, 17600512, 19468925, 9384402, 9192383, 25379886 | | |
| 1.37 Balance beam | **Deficits:** 19998488, 8683262, 23667624, 23743262, 25555930, 25548960, 10595822, 7773695, 12225654, 17188268, 28552947, 16545803, 24231261, 25270295, 24691765, 8411215, 27779341, 23327111, 28685977, 9284075, 23294038, 17828037, 26484783, 25698949, 24283269, 9192383, 27498246, 20621186, 22034986, 16042992, 26258958, 24158195  **No deficits:** 23743262, 17188268, 16545803, 9284075, 23294038 | | |
| 1.38 open field | **Deficits:** 26474659, 22498103, 22652305, 27779341, 7496806, 27899434, 26414556, 28288867, 27988361, 15002855, 26484783, 25698949, 24283269, 25379886, 22034986, 28855139, 26756169, 28685977  **No deficits:** 27899434, 26414556, 28288867, 15002855, 28855139, 10659149 | | |
| 1.39 rotarod | **Deficits** 19998488, 23667624, 28070456, 23743262, 23622728, 24088994, 22424792, 28288867, 28514188, 26258958, 27072754  **No deficits** 28070456, 23743262, 22424792, 28288867, 28514188, 27072754 | | |
| 1.40 Other motor behaviors | **Foot Placement:** 19998488, 23198799, 17828037, 11223919  **Grid Walking:** 28070456  **Grip Test:** 14577862, 21325251, 26458361, 20940727  **Tape Removal:** 22034986  **General Activity:** 19605168, 15289185, 26448536  **Staircase Test:** 21120456  **Whisker Test:** 26258958, 27072754  **Seizure Susceptibility:** 26537880  **Thermal or Mechanical withdrawal:** 28321191 | | |
| 1.41 Barnes maze | **Deficits:** 10.4274/haseki.3446, 28855139 | | |
| 1.42 Radial arm water maze | **Deficits:** 23354401, 27357503 | | |
| 1.43 y/t maze | **Deficits:** 25879458, 21740439, 25106629, 27285176, 28690173, 20001584, 25124230, 21120456, 28514188, 11223919, 17669633  **No deficits:** 28855139, 10659149 | | |
| 1.44 novel object | **Deficits:** 25879458, 21740439, 23743262, 25555930, 23178198, 10547099, 23622728, 28284950, 25394735, 22652305, 18260794, 23040263, 23059457, 28690173, 20001584, 27899434, 26414556, 25124230, 21120456, 28514188, 25698949, 11223919, 17351125, 21910642, 7583233, 16099948, 23184513  **No deficits:** 21770756, 26528156, 10659149 | | |
| 1.45 morris water maze | **Deficits:** 7773695, 24938400, 20951803, 15289185, 25872478, 28194658, 18407501, 16084512, 10082192, 27923162, 20940727, 26474659, 28284950, 25394735, 25270295, 24239694, 22120305, 9284075, 21120456, 23294038, 26709201, 29499284, 25698949, 24283269, 27498246, 27373531, 12732240, 16356639, 21499325  **No deficits:** 25555930, 25548960, 28194658, 21120456, 16356639 | | |
| 1.46 Fear conditioning | **Deficits:** 27899434, 10.5812/archneurosci.38039  **No deficits:** 22498103, 28321191 | | |
| 1.47 passive avoidance | 24231261, 28321191 |  | |
| 1.48 Other cognitive | **Water finding:** 9062682, 9062683, 9313933, WOS:A1997YK51100004  **Location discrimination:** 28558476  **Go/no-go testing:** 26448536  **Closed circle exiting:** 24158195  **Novel Context mismatch:** 26709201 | | |
| 1.49 Social | **Deficits**: 24231261, 28321191 |  | |
| 1.50 Elevated plus maze | **Deficits:** 25555930, 25548960, 23178198, 27363926, 27285176, 25698949, 28855139, 7583233  **No deficits:** 25879458, 21740439, 28284950, 22652305, 21120456, 28514188 | | |
| 1.51 other affective behaviors | **Temperature sensitivity:** 26972829  **Tail suspension:** 26474659, 28284950, 22652305  **Olfactory avoidance:** 21910642  **Nociception:** 25168788, 21120456  **Forced swim:** 15815471, 28514188, 25698949, 28855139, 28321191, 24957202, 7583233  **Fear conditioning:** 15002855  **Acoustic startle reflex:** 25379886 | | |
| 1.52 cellular changes | **Deficits:** 25879458, 21740439, 25168788, 10.5336/medsci.2009-11356, 24810171, 25438952, 16758355, 9204117, 10447072, 10101254, 11744229, 26972829, 28461271, 15972243, 8121612, 17289399, 19812968, 8014208, 1295574, 7773695, 9112087, 12225654, 25873133, 10391365, 23622728, 20493841, 19958766, 11686497, 27602324, 9462472, 26124685, 25872478, 27615406, 18407501, 15036604, 10.4274/haseki.3446, 27923162, 16545803, 16842619, 28214984, 18240300, 11706345, 29665077, 21904255, 19133139, 9062683, 19468925, 28659494, 26414556, 9284075, 27498246, 11223919, 15902197, 27357503, 23046422, 23184513, 25450468  **No deficits:** 17612919, 15168230, 9785592, 26157981, 29746557, 8283270, 22498103, 23040263, 18651249, 7652031, 17828037, 10.5812/archneurosci.38039, 26868732, 22120305 | | |
| 1.53 Axonal Injury | **Deficits:** 25168788, 15168230, 14521991, 11744229, 7968229, 17289399, 26157981, 26458361, 10208272, 11686497, 22424792, 15289185, 29746557, 25290011, 19660237, 15036604, 28552947, 28284950, 19688182, 21770756, 21904255, 10996218, 23327111, 9421456, 9062683, 26414556, 26912636, 25770855, 26484783, 27498246, 23667624, 26868732, 9433909, 27266706, 20940727, 25450468  **No deficits:** 21740439, 17828037 | | |
| 1.54 BBB Disruption | 10447072, 25081505, 11435946, 8121612, 8014208, 7773695, 23884669, 9119900, 8317722, 22335783, 27188340, 29330386, 20507613, 21499325, 14577862, 25450468 | | |
| 1.55 Edema | 12657997, 12589996, 7976618, 10494671, 27521753, 24387843, 23262351, 9620509, 23622728, 19484631, 23608674, 8411215, 7976614, 27155455, 12225655, 29330386 | | |
| 1.56 gliosis | 15254773, 16004578, 28070456, 23743262, 25081505, 14659826, 26157981, 23622728, 20493841, 11686497, 22569191, 9462472, 17188268, 25872478, 29746557, 8283270, 29463176, 28552947, 11780864, 11336441, 11526986, 27779341, 21904255, 19133139, 11450053, 28685977, 22335783, 28659494, 21871613, 9284075, 11083921, 25770855, 28274861, 15902197, 27230970, 17351125, 27072754, 28855139, 10.5812/archneurosci.38039, 26868732, 23184513, 27266706, 20940727, 25450468 | | |
| 1.57 Myelin | 11744229, 20493841, 29746557, 25290011, 27498246, 27230970 | | |
| 1.58 Other histology | **TUNEL:** 24810171, 16004578, 21325251, 21466884, 8121612, 17188268, 28214984, 22498103, 10698075, 16960300, 22120305, 16099948  **Inflammation:** 20850781, 14659826, 24088994, 22569191, 26275125, 19133139, 28659494, 27547849, 22173016, 20940727  **Caspase:** 16758355, 29367701, 17188500  **Complement:** 28383046, 17474994, 10698075 | | |
| Piston Driven models | | | |
| 2.1 Species | **Mice:** 25886256, 26806371, 17610350, 18590543, 11782990, 21299360, 18440507, 26541248, 27657499, 16455070, 29665077, 23984869, 27188531, 16960300, 20486807, 25241288, 20812776, 15698624, 28274861, 23443171, 25025304, 26529240, 27357503, 20507613, 17521631, 27845195, 25668562, 25904805, 26153729, 29739276, 27540748, 27656033  **Rats:** 22149927, 10.1001/archneurpsyc.1941.02280130011001, 16822489, 5091699, 29648487, 28038986, 26709201, 18702633, 24283269, 27984100, 874548 | | |
| 2.2 Sex | **Males:** 25886256, 26806371, 17610350, 18590543, 11782990, 8622802, 22149927, 18447626, 17264861, 23198799, 5091699, 21299360, 27657499, 16455070, 23808389, 20486807, 25241288, 20812776, 15698624, 18702633, 17521631, 25668562, 11931348, 24158195, 27540748, 27984100, 874548  **Females:** 27845195 | | |
| 2.3 males and females | **Analyzed by sex** 27365176, 29648487, 28038986, 28988852, 26484783, 27357503, 27845195, 26153729, 27656033  **Did not analyze by sex:** 27365176, 16822489, 18440507, 29648487, 26541248, 28038986, 27188531, 28988852, 26484783, 25025304, 27357503, 27845195, 25904805, 26153729, 29739276, 27656033 | | |
| 2.4 Age | **Adult:** 25886256, 26806371, 17610350, 18590543, 11782990, 8622802, 22149927, 18447626, 10.1001/archneurpsyc.1941.02280130011001, 17264861, 23198799, 5091699, 21299360, 27657499, 16455070, 20138993, 20486807, 26709201, 25241288, 20812776, 15698624, 18702633, 24283269, 27373531, 17521631, 27845195, 25668562, 24158195, 25904805, 26153729, 27540748, 27656033, 27984100, 874548  **Young:** 16822489, 18440507, 29648487, 26541248, 28038986, 29665077, 23984869, 29739276 | | |
| 2.5 Scalp | 27365176, 29648487, 27657499, 29665077, 23443171, 27540748, 874548 | | |
| 2.6 skull | 25886256, 26806371, 11782990, 874550, 16822489, 21299360, 18440507, 26541248, 28038986, 23984869, 27188531, 28274861, 25025304, 26529240, 27357503, 27845195, 25668562, 25904805, 26153729, 29739276, 27656033 | | |
| 2.7 scalp + Helmet | 10.1001/archneurpsyc.1941.02280130011001, 5091699, 24283269, 27984100 | | |
| 2.8 Skull + Helmet | 17610350, 18590543, 16455070, 16960300, 20486807, 25241288, 20812776, 15698624, 18702633, 20507613, 17521631 | | |
| 2.9 head not secured | 26806371, 8622802, 22149927, 10.1001/archneurpsyc.1941.02280130011001, 29648487, 27188531, 26709201, 25241288, 18702633, 24283269, 11931348, 29739276, 27984100, 874548 | | |
| 2.10 animal on a foam pad | 26806371, 18702633, 25025304, 29739276 | | |
| 2.11 animal on foil | 29648487, 29665077 | | |
| 2.12 animal fixed in ear bars | 25886256, 18590543, 11782990, 16822489, 18440507, 28038986, 23984869, 27188531, 16960300, 20812776, 26529240, 27357503, 20507613, 17521631, 27845195, 25668562, 25904805, 26153729, 27540748, 27656033 | | |
| 2.13 mice, impact velocity | **6.8 m/s:** 16455070, 16960300, 25241288, 20812776, 15698624, 17521631  **5 m/s:** 25886256, 21299360, 26541248, 26529240, 25904805, 29739276, 27540748 | | |
| 2.14 rats, impact velocity | **5-10 m/s:** 27365176, 874549, 874550, 28038986, 28988852 | | |
| 2.15 3.2 cm head displacement, mice | 25886256, 26806371, 17610350, 18590543, 11782990, 21299360, 18440507, 26541248, 27657499, 16455070, 29665077, 23984869, 27188531, 16960300, 20486807, 25241288, 15698624, 25025304, 26529240, 27357503, 20507613, 27845195, 25668562, 25904805, 26153729, 29739276, 27540748, 27656033 | | |
| 2.16 Tip material | **Rigid:** 25886256, 17610350, 18590543, 8622802, 18447626, 17264861, 23198799, 21299360, 18440507, 29648487, 26541248, 28038986, 16455070, 23808389, 27188531, 20486807, 20812776, 15698624, 18702633, 25668562, 25904805, 26153729, 29739276, 27984100, 874548  **Non-rigid:** 26806371, 5091699 | | |
| 2.17 Tip material not reported | 11782990, 22149927, 10.1001/archneurpsyc.1941.02280130011001, 16822489, 27657499, 20138993, 26709201, 25241288, 24283269, 27373531, 17521631, 27845195, 11931348, 24158195, 27540748, 27656033 | | |
| 2.18 impact tip shape | **Flat:** 25886256, 23198799, 18440507, 23808389, 20486807, 25241288, 27373531, 25668562, 25904805, 27540748, 27656033, 27984100  **Round** 21299360, 26541248, 29665077, 26529240, 27845195, 26153729, 29739276 | | |
| 2.19 mortality | **Low (0-5%):** 25886256, 5091699, 21299360, 29648487, 27657499, 23984869, 23443171, 25668562, 25904805  **Moderate (5-30%)** 27656033 | | |
| 2.20 righting reflex | **1-10 minutes:** 8622802, 10.1001/archneurpsyc.1941.02280130011001, 21299360, 27657499, 28038986, 26709201, 24283269, 25668562, 24158195, 25904805, 27984100  **10+ minutes** 27188531 | | |
| 2.21 Balance beam | **Deficits:** 27365176, 26541248, 28988852, 26484783, 25025304  **No deficits:** 27656033 | | |
| 2.22 rotarod | **Deficits** 17610350, 27657499, 16455070, 25241288, 20812776, 28274861, 23443171, 17521631, 27656033  **No deficits:** 15698624 | | |
| 2.23 open field | **Deficits:** 27365176, 29665077, 28988852, 26484783, 27357503  **No deficits:** 27656033 | | |
| 2.24 other motor behaviors | **Wheel Running:** 25904805  **Wire hanging:** 23984869 | | |
| 2.25 Morris water maze | **Deficits:** 17610350, 11782990, 23198799, 21299360, 28038986, 25241288, 18702633  **No deficits:** 16455070 | | |
| 2.26 Other cognitive testing | **Fear conditioning:** 25025304, 26529240, 27984100  **Novel object:** 27365176, 26484783, 23443171  **Y/T maze:** 18590543, 29665077  **Radial arm water maze:** 25886256, 25904805  **Barnes maze:** 20486807, 23443171  **Passive avoidance:** 27845195  **Labrynith:** 5091699 | | |
| 2.27 elevated plus | **Deficits:** 10.1001/archneurpsyc.1941.02280130011001, 26709201, 24283269  **No deficits:** 8622802 | | |
| 2.28 Social testing | **Deficits:** 10.1001/archneurpsyc.1941.02280130011001, 26709201, 24283269  **No deficits:** 8622802 | | |
| 2.29 cell morphology chAnges | **Deficits:** 17610350, 11782990, 23198799, 21299360, 26541248, 28038986, 25241288, 20812776, 18702633  **No deficits:** 20486807 | | |
| 2.30 Axonal injury | **Deficits:** 10.1001/archneurpsyc.1941.02280130011001, 17264861, 16822489, 21299360, 18440507, 27657499, 28038986, 27188531, 20138993, 20812776, 27845195, 25668562, 26153729  **No deficits:** 18447626 | | |
| 2.31 gliosis | **Deficits:** 25886256, 26806371, 17610350, 8622802, 18447626, 17264861, 23198799, 26541248, 28038986, 27188531, 20138993, 20486807, 20812776, 27373531, 27845195, 25668562, 11931348, 25904805, 27656033 | | |
| 2.32 myelin | **Deficits:** 17264861, 26541248, 25668562 | | |
| 2.33 Other histology | **Blood brain barrier:** 23984869, 23443171  **Inflammation:** 18590543, 25241288  **Caspase activation:** 18440507  **Complement activation:** 20812776 | | |
| ‘other’ models | | | |
| 3.1 rotational models |  | |  |
|  | **MCW Rotational Model:** 15850087, 25344352, 27188340, 16817594 | | |
|  | **Rostami Model:** 23808389, 22837752 | | |
| 3.2 Anesthesia | **Yes:** 21988140, 9361992, 2444951, 24756867, 27521753, 25957560, 5170668, 27167127, 8121612, 3416806, 15850087, 17711400, 27138134, 19929375, 23808389, 25344352, 7723065, 22837752, 27188340, 28780369  **No:** 458474, 3416806, 6093755, 19723705, 26529240 | | |
| 3.3 head fixed | **Yes:** 21988140, 9361992, 24756867, 27521753, 25957560, 27167127, 8121612, 4070440, 3016201, 3416806, 6093755, 15850087, 17711400, 27138134, 19929375, 23808389, 25344352, 7723065, 22837752, 27188340, 28780369  **No:** 458474, 2444951, 16689671, 26529240 | | |
| 3.4 Scalp + helmet | 21988140, 24756867, 15850087, 17711400, 25344352, 27188340 | | |
| 3.5 skull + Helmet | 5170668, 23808389, 22837752 | | |
| 3.6 scalp | 9361992, 458474, 2444951, 10.1001/archneurpsyc.1941.02280130011001, 8121612, 4070440, 3016201, 3416806, 6093755, 7723065, 28780369 | | |
| 3.7 skull | 27521753, 25957560, 27167127, 27138134, 19929375 | | |
| 3.8 Species | **Rat:** 21988140, 9361992, 458474, 2444951, 24756867, 5170668, 10.1001/archneurpsyc.1941.02280130011001, 8121612, 4070440, 3016201, 3416806, 6093755, 15850087, 17711400, 27138134, 19929375, 25344352, 7723065, 22837752, 27188340, 28780369  **Mice:** 25957560, 25982185, 23443171 | | |
| 3.9 sex | **Male:** 21988140, 9361992, 458474, 2444951, 24756867, 25957560, 5170668, 4070440, 3016201, 3416806, 6093755, 15850087, 17711400, 27138134, 19929375, 23808389, 7723065, 22837752  **Female:** 10899295, 25344352, 28780369 | | |
| 3.10 Balance beam | **Deficit:** 21988140, 24756867, 7723065  **No deficit:** 19929375, 22837752 | | |
| 3.11 Rotarod | 21988140 | | |
| 3.12 other behavioral | **Incline plane:** 7723065  **Gait analysis:** 24756867  **Vision changes:** 15850087 | | |
| 3.13 Cognitive | **Morris water maze:** 21988140, 25344352, 27188340, 28780369  **Radial arm water maze:** 22837752  **Novel object recognition;** 28780369 | | |
| 3.14 Elevated plus | 25344352, 22837752, 27188340 | | |
| 3.15 Cell morphology | **Deficit:** 25957560, 27167127, 10.1001/archneurpsyc.1941.02280130011001, 15850087, 17711400, 23808389  **No deficit:** 21988140 | | |
| 3.16 axonal injury | **Deficit:** 24756867, 19929375, 23808389, 7723065, 22837752  **No deficit:** 21988140, 17711400 | | |
| 3.17 gliosis | **Deficit:** 21988140, 24756867, 25957560, 27167127  **No deficit:** 27138134 | | |
| 3.18 other histology | **Complement activation:** 23808389  **Doublecortin:** 28780369  **BDNF:** 28780369  **Aquaporin-4:** 25957560 | | |
